# Statistical analysis plan for a cluster randomised trial in Madhya Pradesh, India: community health promotion and medical provision and impact on neonates (CHAMPION2)

**DOI:** 10.1186/s13063-024-08056-2

**Published:** 2024-04-25

**Authors:** Nicholas Magill, Siddharudha Shivalli, Ila Fazzio, Diana Elbourne, Suzanne Keddie, Padmanabh Reddy, Rakhi Nair, Madan Gopal, Sridevi Karnati, Harshavardhan Reddy, Peter Boone, Chris Frost

**Affiliations:** 1 London School of Hygiene and Tropical Medicine, London, UK; 2 Effective Intervention, London, UK; 3 NICE Foundation, Hyderabad, India; 4 GH Training and Consulting, Hyderabad, India

## Abstract

**Background:**

Neonatal mortality in India has fallen steadily and was estimated to be 24 per 1000 live births in the year 2017. However, neonatal mortality remains high in rural parts of the country. The Community Health Promotion and Medical Provision and Impact On Neonates (CHAMPION2) trial investigates the effect of a complex health intervention on neonatal mortality in the Satna District of Madhya Pradesh.

**Methods/design:**

The CHAMPION2 trial forms one part of a cluster-randomised controlled trial with villages (clusters) randomised to receive either a health (CHAMPION2) or education (STRIPES2) intervention. Villages receiving the health intervention are controls for the education intervention and vice versa. The primary outcome is neonatal mortality. The effect of the active intervention on the primary outcome (compared to usual care) will be expressed as a risk ratio, estimated using a generalised estimating equation approach with robust standard errors that take account of clustering at village level. Secondary outcomes include maternal mortality, stillbirths, perinatal deaths, causes of death, health care and knowledge, hospital admissions of enrolled women during pregnancy or in the immediate post-natal care period or of their babies (during the neonatal period), maternal blood transfusions, and the cost effectiveness of the intervention. A total of 196 villages have been randomised and over 34,000 women have been recruited in CHAMPION2.

**Discussion:**

This update to the published trial protocol gives a detailed plan for the statistical analysis of the CHAMPION2 trial.

**Trial registration:**

Registry of India: CTRI/2019/05/019296. Registered on 23 May 2019. https://ctri.nic.in/Clinicaltrials/pmaindet2.php?EncHid=MzExOTg=&Enc=&userName=champion2

**Supplementary Information:**

The online version contains supplementary material available at 10.1186/s13063-024-08056-2.

## Introduction

### Background and rationale

Estimates from 2019 suggest that 2.4 million neonatal deaths occur annually, of which about 20% (522,000) are in India [1]. In 2015, the major determinants of neonatal deaths in India were prematurity and low birth weight (44%), birth asphyxia and birth trauma (19%), and neonatal infections (19%) [2]. Despite India’s rapid economic growth, most Indian states have exhibited a slower than hoped decline in neonatal mortality rates from 2000 to 2015 [2]. India’s neonatal mortality rate (NMR) reduced from 38 to 24 per 1000 live births from 2000 to 2017 [3]. Estimates vary widely between the different states, e.g. the NMR is 5 per 1000 live births in Kerala and 33 per 1000 live births in Madhya Pradesh [4]. There are large disparities in health, with NMRs almost twice higher in rural areas compared to urban areas (14 and 27 per 1000 live births in urban and rural areas, respectively) [4]. The state of Madhya Pradesh is characterised by a marginalised, tribal population, where less than 30% of mothers in rural villages have four or more antenatal care visits and the proportion of illiterate women is about 50% [5]. Within the state, the NMR ranges between 24 per 1000 live births in Indore district and 57 per 1000 live births in Satna district [6]. The original CHAMPION trial in Telangana compared an intervention aimed at reducing neonatal mortality to a control arm offering the usual ongoing health services. The health intervention included a package comprising community health promotion (health education through village health worker-led participatory discussion groups), outreach (mobile teams providing antenatal and postnatal care in the home or through fixed day health services), and provision of facility-based care (subsidised access to non-public health centres) [7]. The primary outcome of neonatal mortality was significantly lower in the intervention arm compared to the control arm (52 neonatal deaths per 1000 live births versus 69 deaths per 1000 live births), a reduction of 24% (relative risk 0.76; 95% CI 0.64 to 0.90; *p* = 0.0018). The CHAMPION2 trial investigates whether an adapted intervention will have a similar effect on neonatal mortality in Satna district of Madhya Pradesh, India [8].

### Objectives

The objective of the CHAMPION2 trial is to assess whether the success of the CHAMPION trial in providing community health promotion, outreach, and provision of facility-based care and in reducing neonatal mortality can be replicated in Satna district of Madhya Pradesh, India.

The primary outcome is neonatal mortality. Secondary outcomes include maternal mortality, stillbirths and perinatal deaths, causes of death, antenatal care, delivery care, immediate neonatal care, postnatal care, health knowledge, hospital admissions of enrolled women during pregnancy or afterwards or of their babies (during the neonatal period), maternal blood transfusions, and the cost effectiveness of the intervention.

## Study methods

### Trial design

The CHAMPION2 trial forms one part of a cluster-randomised controlled trial with villages (clusters) in the Satna district of Madhya Pradesh, India, randomised to receive either a health (CHAMPION2) or education (STRIPES2- support to rural India’s public education system and impact on numeracy and literacy scores) intervention. Building on the design of the earlier CHAMPION/STRIPES trial, villages receiving the health intervention are controls for the education intervention and vice versa [7].

Villages were potentially eligible if the following conditions were met:

1. A village in Satna district, except villages in the tehsils of: Birsinghpur, Majhgawan, and Raghurajnagar;
2. The village was considered rural with a population less than 2500;
3. The village has more than 120 children under the age of 6 and at least 15 children eligible for the STRIPES2 intervention;
4. The village is accessible by road;
5. The village centre is at least 5 km from all community health centres and civil hospitals (as such villages are already well-served by the local health services);
6. The village centre is at least 3 km from the centre of any other included village, with this criterion included to minimise contamination.

At the time of the initial identification of eligible villages, we did not take account of the location of one civil hospital. We subsequently discovered that one of our selected villages was less than 5 km (but more than 4 km) from this civil hospital. Since the village had already been informed about the trial, we decided that the village should be retained and randomised.

### Randomisation

Randomisation of clusters was performed by the senior trial statistician based in London in June 2019 using a random number generator, with stratification by village size (less than or greater than the median) and distance to the nearest community health centre or civil hospital (less than or greater than the median).

### Sample size

The relevant parts of the original sample size calculation as published in the protocol were as follows.

After clusters (villages) were identified, there were 484 villages that were potentially eligible for the trial. Originally, it had been the intention to randomise 300 villages, because this gave over 90% statistical power to detect (1) a 20% reduction in neonatal mortality in CHAMPION2 and (2) a difference of 0.25 in mean test scores, when standardised to have unit standard deviation, in STRIPES2. However, incorporating the buffer zones described in the village selection procedure as described previously meant that only 204 villages could be selected. These 204 villages have a mean population of 1487 (minimum 558, maximum 2490) and SD of 505 (equating to a coefficient of variation of 0.34). Estimating the number of children in each school year from the number younger than 6 years (divided by 6), the mean number of children in each school year is 38.3 (minimum 20, maximum 71) with SD of 13.3 (coefficient of variation 0.35). Assuming that 25% of the children would not be eligible according to the criteria, this gave an estimated mean number of eligible children per village of 28.7 with a minimum of 15.

In CHAMPION, the design effect for neonatal mortality was 1.306, equating to an intra-cluster correlation coefficient (ICC) of 0.011 (with allowance for variability in cluster size, the assumed coefficient of variation = 0.34). For CHAMPION2, allowing for the fact that each village has an average population of 1487 and estimated crude birth rate of 30.7 per 1000 population per year in rural areas of Satna district [4], 114 births per village over a 30-month follow-up period were expected. Assuming (1) an ICC of 0.011 for the primary outcome, (2) an assumed coefficient of variation for village size variability of 0.34 (3), that 5% of villages would be excluded for reasons such as withholding consent, and (4) that there would be 10% loss to follow-up, a trial with 194 villages (95% of 204) had 75% power (5% two-sided significance) to detect a 20% reduction in neonatal mortality from 6.7% to 5.36% and 91% power (5% 2-sided significance) to detect a 25% reduction in neonatal mortality from 6.7% to 5.0% (this and other power and sample size calculations were performed using the “clustersampsi” command in Stata 14) [9]. Since the reduction in neonatal mortality seen in CHAMPION was 25%, proceeding with 204 villages seemed reasonable given the requirement for buffer zones in order to avoid contamination. In fact, 196 were randomised, with six villages removed since they were found to be too close to urban areas to be considered rural, and two removed because insufficient eligible children for STRIPES2 were found.

### Framework

The trial will use a superiority hypothesis testing framework.

### Statistical interim analyses and stopping guidance

CHAMPION2 interim analyses were pre-specified and provided confidentially by the trial statisticians to an independent data monitoring committee (DMC), which was guided by the Peto-Haybittle rule [10] with a recommendation to stop made on the basis of a large and statistically significant (*p* < 0.001) difference in either direction. The DMC met twice and reported to the trial steering committee (TSC).

Under the Peto-Haybittle rule, a recommendation is made to stop the trial if an interim analysis shows strong evidence of an effect (*p* < 0.001). One consequence of setting the threshold for stopping so high is that the significance level of the final analysis can remain at 0.05 without a material increase in the overall chance of a false positive.

### Timing of final analysis

January 2024 to March 2024.

### Timing of outcome assessments

The analysis included data relating to births that occurred between 1 January 2021 and 30 June 2023.

Neonatal mortality and stillbirths were assessed from 24 h after delivery and in the following weeks. If the baby was less than 28 days at the time of assessment and alive, there was another assessment after the baby had completed 28 days of life.

Maternal mortality was assessed as part of the routine monthly data collection and monitoring of all enrolled women. Whenever the monitoring system recorded a woman as having died and also recorded that the woman had either reported a pregnancy or had a delivery in the previous 2 months, a verbal autopsy was conducted, and medical consultants assessed whether this was a maternal death occurring within 42 days of delivery and assigned the cause of death.

After the neonatal period (29 days or more after the delivery), whenever the mother was available and willing to be interviewed, we collected the following secondary outcomes: antenatal care, delivery care, immediate neonatal care, postnatal care, and health knowledge. These data were collected for all cases of neonatal death and stillbirths, all multiple births, and a random sample of 10% of other live neonates.

Hospitalisation and blood transfusion during pregnancy were collected for all mothers whose pregnancy ended at or after 28 weeks gestation. For the period of maternal postnatal care, we collected information only for the immediate postnatal period (i.e. 24 h post delivery) and where there was a maternal death. Hospitalisation of babies was assessed during the immediate postnatal period (for the first 24 h of life) and where there was a neonatal death. Blood transfusion was assessed after the neonatal period only where there was a neonatal death.

Adherence to intervention was assessed throughout pregnancy and the neonatal period by recording women’s attendance at services offered as part of the intervention (see the “Definition of adherence to the intervention and how this is assessed including extent of exposure” section).

## Statistical principles

### Level of statistical significance

5%.

### Adjustments for multiplicity

None (not applicable)

### Confidence intervals to be reported

Yes, 95% confidence intervals.

### Definition of adherence to the intervention and how this is assessed including extent of exposure

The intervention is described in the protocol [8]. In brief, it comprised community health promotion, community mobilisation with women’s groups, the provision of fixed-day services, and facilitation of referrals of mothers and neonates to community health centres or civil hospitals. The first of these elements was a health promotion campaign to promote health knowledge relating to maternal and neonatal health. Community mobilisation was delivered with women’s groups in the form of participatory learning and action (PLA). These group sessions featured discussions about issues related to maternal and neonatal health. The fixed-day services consisted of mobile teams providing a package of antenatal care (ANC) and postnatal care (PNC). Finally, the intervention included the facilitation and monitoring of referrals of mothers and neonates to the nearest community health centre or civil hospital. See Table 1 for summaries of attendances at PLA, ANC and PNC sessions.

**Table 1:** Adherence to the intervention (number (%) unless otherwise stated)

	CHAMPION2 intervention arm
**Counting pregnancies**	*x*
**No documented care delivered by the NICE Foundation**	*x* (*x*)
**Documented care delivered by the NICE Foundation**	*x* (*x*)
**Participatory learning and action sessions attended where there is documented care by the NICE Foundation**	
0	*x* (*x*)
1	*x* (*x*)
2	*x* (*x*)
3	*x* (*x*)
4–7	*x* (*x*)
8–11	*x* (*x*)
12–15	*x* (*x*)
16+	*x* (*x*)
**Antenatal care sessions attended where there is documented care by the NICE Foundation**	
0	*x* (*x*)
1	*x* (*x*)
2	*x* (*x*)
3	*x* (*x*)
4–7	*x* (*x*)
8–11	*x* (*x*)
12–15	*x* (*x*)
16+	*x* (*x*)
**Postnatal care sessions attended where there is documented care by the NICE Foundation**	
0	*x* (*x*)
1	*x* (*x*)
2	*x* (*x*)
3	*x* (*x*)
4	*x* (*x*)
5+	*x* (*x*)
**Pregnancy with adequate adherence to intervention**^**a**^	
Yes	*x* (*x*)
No	*x* (*x*)

^a^Adequate adherence is defined as attendance of three or more PLA sessions, four or more ANC sessions, and three or more PNC sessions by mothers of babies who survived the first 28 days of life (i.e. the neonatal period). For mothers of babies who died, adequate adherence is defined as attendance of three or more PLA sessions and four or more ANC sessions

For pregnancies where the baby survived the neonatal period (the first 28 days of life), adequate adherence to the intervention is defined as attendance at three or more PLA sessions, four or more ANC sessions, and three or more PNC sessions. For pregnancies where the neonate died, adequate adherence is defined as attendance of three or more PLA sessions and four or more ANC sessions. The per-protocol population will be defined as those pregnancies with adequate adherence as defined here.

### Definition of protocol deviations for the trial

Deviation from the protocol is defined as either (1) an intervention village not receiving any of the intervention during the trial intervention period or (2) a control village receiving any component of the intervention during the trial intervention period. Such protocol deviations will be listed.

### Analysis populations

The trial includes a number of analysis populations. These are defined as follows:

*Eligible women population (population W1)*: A woman was eligible for CHAMPION2 if during enumeration (pre-randomisation) she satisfied all the following criteria:

1. She was married,
2. Neither she nor her husband had a family planning operation (i.e. tubectomy or vasectomy),
3. She was younger than 50 years of age,
4. She was resident of one of the trial villages at the time of the baseline survey and
5. She gave her consent after being given a complete explanation of the study.

In addition, a woman was eligible if she married a man who was enumerated and unmarried at the time of enumeration, resident of the village, and aged between 13 and 50 years. If an eligible woman died, her widowed husband was added to the list of unmarried men. If this widowed man married again, then his wife was considered eligible. The woman had to fulfil the usual criteria for eligibility including being younger than 50 years of age, resident of the village, gave her consent (this now being post-randomisation), and neither she nor her husband have had a family planning operation.

*Population W2*: Eligible women with at least one pregnancy that went beyond 28 weeks gestation and ended on or after 1 January 2021.

*Population W3*: Eligible women giving birth to a live-born on or after 1 January 2021.

*Population P1*: Pregnancies that went beyond 28 weeks gestation and ended on or after 1 January 2021.

*Eligible children population (population C1)*: all biological children (live-born and still births where the pregnancies went beyond 28 weeks gestation) born to eligible women on or after 1 January 2021.

*Population C2*: All eligible children with adequate adherence to the intervention as defined above for this pregnancy.

*Population C3*: All eligible children who were born alive.

*Population C4:* All eligible children who were born alive and with adequate adherence to the intervention as defined above for this pregnancy.

Populations W1 to W3 are at the woman level, P1 is at the pregnancy level (each woman might have more than one pregnancy), and C1 to C4 are at the child level (each pregnancy might result in more than one born child).

The primary analysis will follow the intention-to-treat principle. For the primary outcome, a secondary per-protocol analysis will be performed using C4.

## Trial population

### Eligibility criteria

The eligibility criteria for villages are described in the “Trial design” section. The eligibility criteria for women and children are described in the “Analysis populations” section.

### Recruitment information to be included in the CONSORT flow diagram Fig. 1

**Fig. 1:**
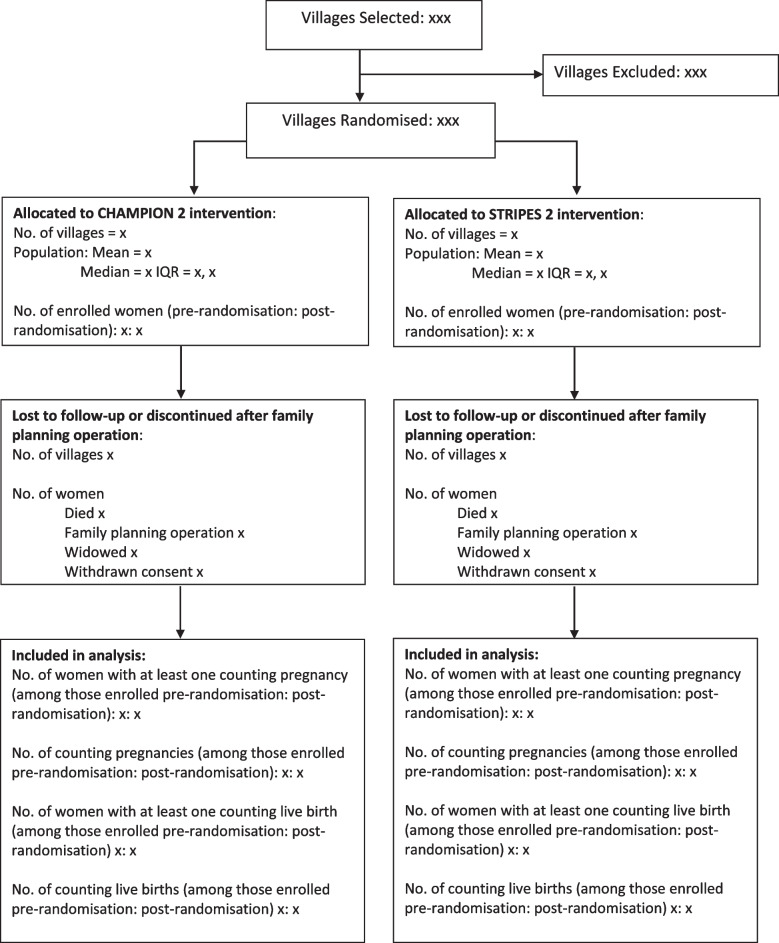
CONSORT flowchart

### Withdrawal/follow-up

As detailed above in the CONSORT flowchart.

### Baseline patient characteristics

The following baseline characteristics will be tabulated by treatment arm. No hypothesis tests comparing baseline characteristics will be carried out. For categorical variables, the overall proportions (with numerators and denominators) will be shown. For continuous variables, the overall mean and standard deviation will be shown.

Cluster-level variables (see Table 2):

- Village size,
- Distance to community health centre/civil hospital,
- Nearest community health centre/civil hospital.

**Table 2:** Baseline characteristics of villages (number (%) unless otherwise stated)

Variable	CHAMPION2 intervention arm (*n* = 98)	STRIPES2 intervention arm (*n* = 98)
**Village size (total population)**		
Mean (SD)	*x* (*x*)	*x* (*x*)
Median (IQR)	*x* (*x*)	*x* (*x*)
0–499 residents	*x* (*x*)	*x* (*x*)
500–1499 residents	*x* (*x*)	*x* (*x*)
≥ 1500 residents	*x* (*x*)	*x* (*x*)
**Distance (km) to nearest civil hospital/community health centre**		
Mean (SD)	*x* (*x*)	*x* (*x*)
Median (IQR)	*x* (*x*)	*x* (*x*)
Greater than median distance (stratification factor)	*x* (*x*)	*x* (*x*)
**Nearest civil hospital/community health centre**		
Amarpatan	*x* (*x*)	*x* (*x*)
Amdara	*x* (*x*)	*x* (*x*)
Devarajnagar	*x* (*x*)	*x* (*x*)
Kothi	*x* (*x*)	*x* (*x*)
Maihar	*x* (*x*)	*x* (*x*)
Mukundpur	*x* (*x*)	*x* (*x*)
Nagod	*x* (*x*)	*x* (*x*)
Ramnagar	*x* (*x*)	*x* (*x*)
Rampur Baghelan	*x* (*x*)	*x* (*x*)
Unchehera	*x* (*x*)	*x* (*x*)

Individual-level variables (see Table 3):

- Age,
- Birth in the year before enrolment,
- Neonatal death in the year before enrolment,
- Pregnant at enrolment,
- Caste,
- Literacy of woman,
- Education level of woman,
- Literacy of husband,
- Education level of husband,
- Wealth index 1. Determined by the material the house is made of: (1) floor, roof, and wall materials all natural; (2) some, but not all, of floor, roof, and wall materials are synthetic; and (3) floor, roof, and wall materials all synthetic (as in [11]),
- Wealth index 2. Number of Items (television, radio, motorbike, 4-wheeled vehicle) owned by the household members.

**Table 3:** Baseline characteristics of all enrolled women (populations W1, W2, W3) (number (%) unless otherwise stated)

	CHAMPION2 intervention arm		All	STRIPES2 intervention arm		All
	Enrolled pre-/post randomisation			Enrolled pre-/post randomisation		
	Pre-	Post-		Pre-	Post-	
**Number of women**	*x* (*x*)	*x* (*x*)	*x* (*x*)	*x* (*x*)	*x* (*x*)	*x* (*x*)
**Age (years)**						
*Mean (SD)*	*x* (*x*)	*x* (*x*)	*x* (*x*)	*x* (*x*)	*x* (*x*)	*x* (*x*)
Women aged:						
15–18	*x* (*x*)	*x* (*x*)	*x* (*x*)	*x* (*x*)	*x* (*x*)	*x* (*x*)
19–29	*x* (*x*)	*x* (*x*)	*x* (*x*)	*x* (*x*)	*x* (*x*)	*x* (*x*)
30–39	*x* (*x*)	*x* (*x*)	*x* (*x*)	*x* (*x*)	*x* (*x*)	*x* (*x*)
40–49	*x* (*x*)	*x* (*x*)	*x* (*x*)	*x* (*x*)	*x* (*x*)	*x* (*x*)
**Caste**						
Scheduled Caste	*x* (*x*)	*x* (*x*)	*x* (*x*)	*x* (*x*)	*x* (*x*)	*x* (*x*)
Scheduled Tribe	*x* (*x*)	*x* (*x*)	*x* (*x*)	*x* (*x*)	*x* (*x*)	*x* (*x*)
Other Backward Caste	*x* (*x*)	*x* (*x*)	*x* (*x*)	*x* (*x*)	*x* (*x*)	*x* (*x*)
Forward Caste	*x* (*x*)	*x* (*x*)	*x* (*x*)	*x* (*x*)	*x* (*x*)	*x* (*x*)
**Education of women**						
No schooling	*x* (*x*)	*x* (*x*)	*x* (*x*)	*x* (*x*)	*x* (*x*)	*x* (*x*)
Primary school	*x* (*x*)	*x* (*x*)	*x* (*x*)	*x* (*x*)	*x* (*x*)	*x* (*x*)
Middle school	*x* (*x*)	*x* (*x*)	*x* (*x*)	*x* (*x*)	*x* (*x*)	*x* (*x*)
High school	*x* (*x*)	*x* (*x*)	*x* (*x*)	*x* (*x*)	*x* (*x*)	*x* (*x*)
Higher secondary school	*x* (*x*)	*x* (*x*)	*x* (*x*)	*x* (*x*)	*x* (*x*)	*x* (*x*)
Graduate	*x* (*x*)	*x* (*x*)	*x* (*x*)	*x* (*x*)	*x* (*x*)	*x* (*x*)
Postgraduate	*x* (*x*)	*x* (*x*)	*x* (*x*)	*x* (*x*)	*x* (*x*)	*x* (*x*)
Not known	*x* (*x*)	*x* (*x*)	*x* (*x*)	*x* (*x*)	*x* (*x*)	*x* (*x*)
**Education of spouse**						
No schooling	*x* (*x*)	*x* (*x*)	*x* (*x*)	*x* (*x*)	*x* (*x*)	*x* (*x*)
Primary school	*x* (*x*)	*x* (*x*)	*x* (*x*)	*x* (*x*)	*x* (*x*)	*x* (*x*)
Middle school	*x* (*x*)	*x* (*x*)	*x* (*x*)	*x* (*x*)	*x* (*x*)	*x* (*x*)
High school	*x* (*x*)	*x* (*x*)	*x* (*x*)	*x* (*x*)	*x* (*x*)	*x* (*x*)
Higher secondary school	*x* (*x*)	*x* (*x*)	*x* (*x*)	*x* (*x*)	*x* (*x*)	*x* (*x*)
Graduate	*x* (*x*)	*x* (*x*)	*x* (*x*)	*x* (*x*)	*x* (*x*)	*x* (*x*)
Postgraduate	*x* (*x*)	*x* (*x*)	*x* (*x*)	*x* (*x*)	*x* (*x*)	*x* (*x*)
Not known	*x* (*x*)	*x* (*x*)	*x* (*x*)	*x* (*x*)	*x* (*x*)	*x* (*x*)
**Literacy**						
Women:						
Cannot read at all	*x* (*x*)	*x* (*x*)	*x* (*x*)	*x* (*x*)	*x* (*x*)	*x* (*x*)
Read only part of the sentence	*x* (*x*)	*x* (*x*)	*x* (*x*)	*x* (*x*)	*x* (*x*)	*x* (*x*)
Read whole sentence	*x* (*x*)	*x* (*x*)	*x* (*x*)	*x* (*x*)	*x* (*x*)	*x* (*x*)
**Literacy**						
Women whose spouse:						
Is not present	*x* (*x*)	*x* (*x*)	*x* (*x*)	*x* (*x*)	*x* (*x*)	*x* (*x*)
Cannot read at all	*x* (*x*)	*x* (*x*)	*x* (*x*)	*x* (*x*)	*x* (*x*)	*x* (*x*)
Read only part of the sentence	*x* (*x*)	*x* (*x*)	*x* (*x*)	*x* (*x*)	*x* (*x*)	*x* (*x*)
Read whole sentence	*x* (*x*)	*x* (*x*)	*x* (*x*)	*x* (*x*)	*x* (*x*)	*x* (*x*)
**Wealth index 1 (materials used in construction of home)**^**a**^						
Category 1	*x* (*x*)	*x* (*x*)	*x* (*x*)	*x* (*x*)	*x* (*x*)	*x* (*x*)
Category 2	*x* (*x*)	*x* (*x*)	*x* (*x*)	*x* (*x*)	*x* (*x*)	*x* (*x*)
Category 3	*x* (*x*)	*x* (*x*)	*x* (*x*)	*x* (*x*)	*x* (*x*)	*x* (*x*)
**Wealth index 2 (items owned)**^**a**^						
0	*x* (*x*)	*x* (*x*)	*x* (*x*)	*x* (*x*)	*x* (*x*)	*x* (*x*)
1	*x* (*x*)	*x* (*x*)	*x* (*x*)	*x* (*x*)	*x* (*x*)	*x* (*x*)
2	*x* (*x*)	*x* (*x*)	*x* (*x*)	*x* (*x*)	*x* (*x*)	*x* (*x*)
3	*x* (*x*)	*x* (*x*)	*x* (*x*)	*x* (*x*)	*x* (*x*)	*x* (*x*)
4	*x* (*x*)	*x* (*x*)	*x* (*x*)	*x* (*x*)	*x* (*x*)	*x* (*x*)
**Women who have previously had a miscarriage/termination/stillbirth**	*x* (*x*)	*x* (*x*)	*x* (*x*)	*x* (*x*)	*x* (*x*)	*x* (*x*)
**Birth in the year before enrolment**						
Yes	*x* (*x*)	*x* (*x*)	*x* (*x*)	*x* (*x*)	*x* (*x*)	*x* (*x*)
No	*x* (*x*)	*x* (*x*)	*x* (*x*)	*x* (*x*)	*x* (*x*)	*x* (*x*)
**Neonatal death (born alive, death within 28 days) in last year**						
Yes	*x* (*x*)	*x* (*x*)	*x* (*x*)	*x* (*x*)	*x* (*x*)	*x* (*x*)
No	*x* (*x*)	*x* (*x*)	*x* (*x*)	*x* (*x*)	*x* (*x*)	*x* (*x*)
**Pregnant at enrolment**						
Yes	*x* (*x*)	*x* (*x*)	*x* (*x*)	*x* (*x*)	*x* (*x*)	*x* (*x*)
No	*x* (*x*)	*x* (*x*)	*x* (*x*)	*x* (*x*)	*x* (*x*)	*x* (*x*)

^a^Not collected for population W1

## Analysis

### Outcomes

The primary outcome of the trial is neonatal mortality, which is defined as the death of a liveborn baby during the first 28 completed days of age (population: C3, with a per-protocol analysis carried out in C4). See Table 4 for summaries of counting 28-week pregnancies, Table 5 for pregnancy outcomes including neonatal mortality, and Table 6 for subgroup analyses (see the “Subgroup analyses” section). Table 7 shows pregnancy outcome by gender.

**Table 4:** Pregnancies (number (%) unless otherwise stated)

	CHAMPION2 intervention arm			STRIPES2 intervention arm		
	Enrolled pre-/post randomisation		All	Enrolled pre-/post randomisation		All
	Pre-	Post-		Pre-	Post	
**Number of women (W1)**	*x*	*x*	*x*	*x*	*x*	*x*
**Age on date of first delivery in trial**^**a**^						
*Mean (SD)*	*x* (*x*)	*x* (*x*)	*x* (*x*)	*x* (*x*)	*x* (*x*)	*x* (*x*)
Women aged:						
15–18	*x* (*x*)	*x* (*x*)	*x* (*x*)	*x* (*x*)	*x* (*x*)	*x* (*x*)
19–29	*x* (*x*)	*x* (*x*)	*x* (*x*)	*x* (*x*)	*x* (*x*)	*x* (*x*)
30–39	*x* (*x*)	*x* (*x*)	*x* (*x*)	*x* (*x*)	*x* (*x*)	*x* (*x*)
40–49	*x* (*x*)	*x* (*x*)	*x* (*x*)	*x* (*x*)	*x* (*x*)	*x* (*x*)
**Number of counting pregnancies**^**b**^						
1	*x* (*x*)	*x* (*x*)	*x* (*x*)	*x* (*x*)	*x* (*x*)	*x* (*x*)
2	*x* (*x*)	*x* (*x*)	*x* (*x*)	*x* (*x*)	*x* (*x*)	*x* (*x*)
3+	*x* (*x*)	*x* (*x*)	*x* (*x*)	*x* (*x*)	*x* (*x*)	*x* (*x*)
**Number of women with at least one counting pregnancy**	*x*	*x*	*x*	*x*	*x*	*x*
**Number of counting pregnancies**	*x*	*x*	*x*	*x*	*x*	*x*
**Number of babies born from counting pregnancies**	*x*	*x*	*x*	*x*	*x*	*x*

^a^Delivery defined here as pregnancy ending at or after 28 weeks gestation

^b^Twins contribute two babies

**Table 5:** Pregnancy outcomes, perinatal mortality, and neonatal mortality (number (%) unless otherwise stated)

	CHAMPION2 intervention arm	STRIPES2 intervention arm	Risk ratio
**Intention to treat**			
**Pregnancy outcome**			
Total	*x*	*x*	
Stillbirth	*x* (*x*)	*x* (*x*)	
Livebirth	*x* (*x*)	*x* (*x*)	
Unknown	*x* (*x*)	*x* (*x*)	
**Outcome of livebirths at 28 days**			
Total	*x* (*x*)	*x* (*x*)	
Neonatal death	*x* (*x*)	*x* (*x*)	
Neonatal death on day of birth (day 0)^a^	*x* (*x*)	*x* (*x*)	
Neonatal death between 1 and 2 days	*x* (*x*)	*x* (*x*)	
Neonatal death between 3 and 6 days	*x* (*x*)	*x* (*x*)	
Neonatal death between 7 and 27 days	*x* (*x*)	*x* (*x*)	
Survived to 28 days	*x* (*x*)	*x* (*x*)	
Unknown	*x* (*x*)	*x* (*x*)	
**Stillbirth rate (stillbirths per 1000 births), ignoring those with unknown status in the denominator**	*x*	*x*	*x*^b^ (95% CI: *x*, *x*)
**Perinatal death rate (deaths per 1000 births), ignoring those with unknown status in the denominator**^**c**^	*x*	*x*	*x*^b^ (95% CI: *x*, *x*)
**Neonatal mortality rate (deaths per 1000 live births), ignoring those with unknown status in the denominator**^**d**^	*x*	*x*	*x*^b^ (95% CI: *x*, *x*)
**Place of birth for babies who died**			
Home	*x* (*x*)	*x* (*x*)	
Hospital/health centre	*x* (*x*)	*x* (*x*)	
On the way to hospital	*x* (*x*)	*x* (*x*)	
Relatives’ home	*x* (*x*)	*x* (*x*)	
**Primary cause of death**^**e**^			
Congenital anomaly	*x* (*x*)	*x* (*x*)	
Birth asphyxia	*x* (*x*)	*x* (*x*)	
Preterm newborn	*x* (*x*)	*x* (*x*)	
Sepsis of newborn	*x* (*x*)	*x* (*x*)	
Others	*x* (*x*)	*x* (*x*)	
Unknown	*x* (*x*)	*x* (*x*)	
**Per-protocol**			
**Pregnancy outcome**			
Total	*x*	*x*	
Stillbirth	*x* (*x*)	*x* (*x*)	
Livebirth	*x* (*x*)	*x* (*x*)	
Unknown	*x* (*x*)	*x* (*x*)	
**Livebirths at 28 days**			
Total	*x* (*x*)	*x* (*x*)	
Neonatal death	*x* (*x*)	*x* (*x*)	
Neonatal death on day of birth (day 0)^a^	*x* (*x*)	*x* (*x*)	
Neonatal death between 1 and 2 days	*x* (*x*)	*x* (*x*)	
Neonatal death between 3 and 6 days	*x* (*x*)	*x* (*x*)	
Neonatal death between 7 and 27 days	*x* (*x*)	*x* (*x*)	
Survived to 28 days	*x* (*x*)	*x* (*x*)	
Unknown	*x* (*x*)	*x* (*x*)	
**Neonatal mortality rate (deaths per 1000 live births), ignoring those with unknown status in the denominator**^**d**^	*x*	*x*	*x*^b^ (95% CI: *x*, *x*)

^a^Note that the date of birth is day 0

^b^From a generalised linear model for a binary outcome with a log link and robust Huber-White standard errors that allow for clustering, with randomisation stratification variables as covariates. See the “Analysis methods” section for details

^c^Numerator is sum of stillbirths and neonatal deaths within 7 days; denominator is sum of stillbirths, neonatal deaths, and those surviving to 28 days

^d^Numerator is neonatal deaths; denominator is sum of neonatal deaths and those surviving to 28 days

^e^Full list of causes to be determined from ICD-11 classification/codes

**Table 6:** Neonatal mortality rate (deaths per 1000 live births) by subgroup, with interaction tests (population C3)

Subgroup	CHAMPION2 intervention arm	STRIPES2 intervention arm	Difference (95% CI)	*p*-value
**Village population**				
Below median	*N*: *x* (*x*)	*N*: *x* (*x*)	*x* (*x*, *x*)	*p = x*
Above median	*N*: *x* (*x*)	*N*: *x* (*x*)	*x* (*x*, *x*)	
**Distance to nearest community health centre/civil hospital**				
Below median	*N*: *x* (*x*)	*N*: *x* (*x*)	*x* (*x*, *x*)	*p = x*
Above median	*N*: *x* (*x*)	*N*: *x* (*x*)	*x* (*x*, *x*)	
**When enrolment took place**				
Pre-randomisation	*N*: *x* (*x*)	*N*: *x* (*x*)	*x* (*x*, *x*)	*p = x*
Post-randomisation	*N*: *x* (*x*)	*N*: *x* (*x*)	*x* (*x*, *x*)	
**Gender**				
Male	*N*: *x* (*x*)	*N*: *x* (*x*)	*x* (*x*, *x*)	*p = x*
Female	*N*: *x* (*x*)	*N*: *x* (*x*)	*x* (*x*, *x*)	
**Caste**				
Scheduled Caste	*N*: *x* (*x*)	*N*: *x* (*x*)	*x* (*x*, *x*)	*p = x*
Scheduled Tribe	*N*: *x* (*x*)	*N*: *x* (*x*)	*x* (*x*, *x*)	
Other Backward Caste	*N*: *x* (*x*)	*N*: *x* (*x*)	*x* (*x*, *x*)	
Forward Caste	*N*: *x* (*x*)	*N*: *x* (*x*)	*x* (*x*, *x*)	
**Wealth index 1 (materials used in construction of home)**				
Category 1	*N*: *x* (*x*)	*N*: *x* (*x*)	*x* (*x*, *x*)	*p = x*
Category 2	*N*: *x* (*x*)	*N*: *x* (*x*)	*x* (*x*, *x*)	
Category 3	*N*: *x* (*x*)	*N*: *x* (*x*)	*x* (*x*, *x*)	
**Wealth index 2 (items owned**)				
0	*N*: *x* (*x*)	*N*: *x* (*x*)	*x* (*x*, *x*)	*p = x (trend test)*
1	*N*: *x* (*x*)	*N*: *x* (*x*)	*x* (*x*, *x*)	
2	*N*: *x* (*x*)	*N*: *x* (*x*)	*x* (*x*, *x*)	
3	*N*: *x* (*x*)	*N*: *x* (*x*)	*x* (*x*, *x*)	
4	*N*: *x* (*x*)	*N*: *x* (*x*)	*x* (*x*, *x*)	
**Female care-giver literacy**				
Cannot read	*N*: *x* (*x*)	*N*: *x* (*x*)	*x* (*x*, *x*)	*p = x*
Can read part of the sentence	*N*: *x* (*x*)	*N*: *x* (*x*)	*x* (*x*, *x*)	
Read entire sentence	*N*: *x* (*x*)	*N*: *x* (*x*)	*x* (*x*, *x*)	
**Male care-giver literacy**				
Cannot read	*N*: *x* (*x*)	*N*: *x* (*x*)	*x* (*x*, *x*)	*p = x*
Can read part of the sentence	*N*: *x* (*x*)	*N*: *x* (*x*)	*x* (*x*, *x*)	
Read entire sentence	*N*: *x* (*x*)	*N*: *x* (*x*)	*x* (*x*, *x*)

**Table 7:** Details of counting children (population C1) (number (%) unless otherwise stated)

	CHAMPION2 intervention arm		STRIPES2 intervention arm	
	Male	Female	Male	Female
**Pregnancy outcome**				
Total	*x* (*x*)	*x* (*x*)	*x* (*x*)	*x* (*x*)
Stillbirth	*x* (*x*)	*x* (*x*)	*x* (*x*)	*x* (*x*)
Livebirth	*x* (*x*)	*x* (*x*)	*x* (*x*)	*x* (*x*)
Unknown	*x* (*x*)	*x* (*x*)	*x* (*x*)	*x* (*x*)
**Length of gestation**				
Less than 37 weeks	*x* (*x*)	*x* (*x*)	*x* (*x*)	*x* (*x*)
37 weeks or more	*x* (*x*)	*x* (*x*)	*x* (*x*)	*x* (*x*)

The following secondary outcomes are to be formally tested and a 95% confidence interval constructed:

- Maternal mortality [12], defined as death of a woman whilst pregnant or within 42 days of termination of pregnancy, irrespective of the duration and site of pregnancy (e.g. fallopian tube, uterus), from any cause related to or aggravated by the pregnancy or its management but not from accidental or incidental causes (see Table 8; population: P1);
- Stillbirths and perinatal deaths [12], with a stillbirth defined as the death of a baby after 28 weeks of pregnancy but before or during birth, and a perinatal death defined as either a stillbirth or the death of neonate before 7 completed days of age (see Table 5; population: C1);
- Hospital admissions (other than for delivery) of enrolled women (during pregnancy and in the immediate post-natal period) and their babies (in the immediate post-natal period) (these are events defined a priori as serious adverse events; see Table 9; populations: P1 for women and C1 for babies);
- Maternal blood transfusion during pregnancy and in the immediate post-natal period (this is one of the events defined a priori as serious adverse events; see Table 9; population: P1);

**Table 8:** Maternal mortality (population P1) (number (%) unless otherwise stated)

	CHAMPION2 intervention arm	STRIPES2 intervention arm	Ratio
**Maternal mortality within 42 days (number dead)**	*x*	*x*	
**Maternal mortality rate (deaths per 100,000 live births)**	*x*	*x*	*x* (95% CI: *x*, *x*)
**Primary cause of death**^**a**^			
Postpartum haemorrhage	*x* (*x*)	*x* (*x*)	
Infection	*x* (*x*)	*x* (*x*)	
Hypertensive disorders	*x* (*x*)	*x* (*x*)	
Others	*x* (*x*)	*x* (*x*)	
Unknown	*x* (*x*)	*x* (*x*)	

^a^Full list of causes to be determined from ICD-11 classification/codes

**Table 9:** Serious adverse events (number (%) unless otherwise stated)

	CHAMPION2 intervention arm	STRIPES2 intervention arm	Risk ratio
**Counting pregnancies**	*x*	*x*	
**Maternal blood transfusion**	*x* (*x*)	*x* (*x*)	*x* (95% CI: *x*, *x*)
Identified during routine follow-up	*x* (*x*)	*x* (*x*)	
Additionally identified at verbal autopsy	*x* (*x*)	*x* (*x*)	
**Mother admitted to hospital**	*x* (*x*)	*x* (*x*)	*x* (95% CI: *x*, *x*)
Identified during routine follow-up	*x* (*x*)	*x* (*x*)	
Identified at verbal autopsy	*x* (*x*)	*x* (*x*)	
Days in hospital in those admitted			
1	*x* (*x*)	*x* (*x*)	
2	*x* (*x*)	*x* (*x*)	
3	*x* (*x*)	*x* (*x*)	
4	*x* (*x*)	*x* (*x*)	
5	*x* (*x*)	*x* (*x*)	
6	*x* (*x*)	*x* (*x*)	
7	*x* (*x*)	*x* (*x*)	
8	*x* (*x*)	*x* (*x*)	
9	*x* (*x*)	*x* (*x*)	
10	*x* (*x*)	*x* (*x*)	
11+	*x* (*x*)	*x* (*x*)	
**Babies from counting pregnancies**	*x*	*x*	
**Baby admitted to hospital**	*x* (*x*)	*x* (*x*)	*x* (95% CI: *x*, *x*)
Identified during routine follow-up	*x* (*x*)	*x* (*x*)	
Identified at verbal autopsy	*x* (*x*)	*x* (*x*)	
Days in hospital in those admitted			
1	*x* (*x*)	*x* (*x*)	
2	*x* (*x*)	*x* (*x*)	
3	*x* (*x*)	*x* (*x*)	
4	*x* (*x*)	*x* (*x*)	
5	*x* (*x*)	*x* (*x*)	
6	*x* (*x*)	*x* (*x*)	
7	*x* (*x*)	*x* (*x*)	
8	*x* (*x*)	*x* (*x*)	
9	*x* (*x*)	*x* (*x*)	
10	*x* (*x*)	*x* (*x*)	
11+	*x* (*x*)	*x* (*x*)	

The following secondary outcomes are to be tabulated but not formally tested:

- Neonatal causes of death (see Table 5; population: C1) and maternal causes of death (see Table 8; population: P1);
- Antenatal and postnatal care of mother (e.g. number of antenatal care visits, antenatal care provided; care provider; see Table 10; population: P1);
- Delivery care (e.g. use of skilled birth attendant, place of delivery, clean delivery practices; see Table 11; population: C1);
- Postnatal care of neonate (e.g. umbilical cord care, thermal care, breastfeeding, care seeking; see Table 11; population: C1);
- Health knowledge (see Table 12; population: P1);
- Cost effectiveness of the intervention.

Summaries of mothers’ antenatal and postnatal care are shown in Table 10. Summaries of delivery, babies’ immediate newborn care, and babies’ care in the first month are shown in Table 11. The data in these two tables were collected for all cases of neonatal death and stillbirths, all multiple births, and a random sample of 10% of other live neonates.

**Table 10:** Mothers’ antenatal and postnatal care (P1) (number (%) unless otherwise stated)

	Mothers of singleton babies who were alive at 28 days		Mothers of multiple birth babies who were all alive at 28 days		Mothers of a baby who was dead at 28 days		Weighted average of mothers of all babies^a^	
	CHAMPION2 intervention arm	STRIPES2 intervention arm	CHAMPION2 intervention arm	STRIPES2 intervention arm	CHAMPION2 intervention arm	STRIPES2 intervention arm	CHAMPION2 intervention arm	STRIPES2 intervention arm
**Antenatal care**								
**Aware of advisability of a minimum of 4 ANC visits during pregnancy**								
No	*x* (*x*)	*x* (*x*)	*x* (*x*)	*x* (*x*)	*x* (*x*)	*x* (*x*)	*x* (*x*)	*x* (*x*)
Yes	*x* (*x*)	*x* (*x*)	*x* (*x*)	*x* (*x*)	*x* (*x*)	*x* (*x*)	*x* (*x*)	*x* (*x*)
Do not know	*x* (*x*)	*x* (*x*)	*x* (*x*)	*x* (*x*)	*x* (*x*)	*x* (*x*)	*x* (*x*)	*x* (*x*)
**Aware of the advisability of a 1st ANC visit within 3 months**								
No	*x* (*x*)	*x* (*x*)	*x *(*x*)	*x* (*x*)	*x* (*x*)	*x* (*x*)	*x* (*x*)	*x* (*x*)
Yes	*x* (*x*)	*x* (*x*)	*x* (*x*)	*x* (*x*)	*x* (*x*)	*x* (*x*)	*x* (*x*)	*x* (*x*)
Do not know	*x* (*x*)	*x* (*x*)	*x* (*x*)	*x* (*x*)	*x* (*x*)	*x* (*x*)	*x* (*x*)	*x* (*x*)
**Had a minimum of 4 ANC visits**								
No	*x* (*x*)	*x* (*x*)	*x* (*x*)	*x* (*x*)	*x* (*x*)	*x* (*x*)	*x* (*x*)	*x* (*x*)
Yes	*x* (*x*)	*x* (*x*)	*x* (*x*)	*x* (*x*)	*x* (*x*)	*x* (*x*)	*x* (*x*)	*x* (*x*)
Do not know	*x* (*x*)	*x* (*x*)	*x* (*x*)	*x* (*x*)	*x* (*x*)	*x* (*x*)	*x* (*x*)	*x* (*x*)
**Had 1st ANC visit within 3 months of pregnancy**								
No	*x* (*x*)	*x* (*x*)	*x* (*x*)	*x* (*x*)	*x* (*x*)	*x* (*x*)	*x* (*x*)	*x* (*x*)
Yes	*x* (*x*)	*x* (*x*)	*x* (*x*)	*x* (*x*)	*x* (*x*)	*x* (*x*)	*x* (*x*)	*x* (*x*)
Do not know	*x* (*x*)	*x* (*x*)	*x* (*x*)	*x* (*x*)	*x* (*x*)	*x* (*x*)	*x* (*x*)	*x* (*x*)
**Taken 100 days of iron and folic acid (IFA) tablets or equivalent** ^**b**^								
No	*x* (*x*)	*x* (*x*)	*x* (*x*)	*x* (*x*)	*x* (*x*)	*x* (*x*)	*x* (*x*)	*x* (*x*)
Yes	*x* (*x*)	*x* (*x*)	*x* (*x*)	*x* (*x*)	*x* (*x*)	*x* (*x*)	*x* (*x*)	*x* (*x*)
Do not know	*x* (*x*)	*x* (*x*)	*x* (*x*)	*x* (*x*)	*x* (*x*)	*x* (*x*)	*x* (*x*)	*x* (*x*)
**Had tetanus toxoid injection**								
No	*x* (*x*)	*x* (*x*)	*x* (*x*)	*x* (*x*)	*x* (*x*)	*x* (*x*)	*x* (*x*)	*x* (*x*)
Yes	*x* (*x*)	*x* (*x*)	*x* (*x*)	*x* (*x*)	*x* (*x*)	*x* (*x*)	*x* (*x*)	*x* (*x*)
Do not know	*x* (*x*)	*x* (*x*)	*x* (*x*)	*x* (*x*)	*x* (*x*)	*x* (*x*)	*x* (*x*)	*x* (*x*)
**Woman had full ANC** ^**c**^								
No	*x* (*x*)	*x* (*x*)	*x* (*x*)	*x* (*x*)	*x* (*x*)	*x* (*x*)	*x* (*x*)	*x* (*x*)
Yes	*x* (*x*)	*x* (*x*)	*x* (*x*)	*x* (*x*)	*x* (*x*)	*x* (*x*)	*x* (*x*)	*x* (*x*)
Do not know	*x* (*x*)	*x* (*x*)	*x* (*x*)	*x* (*x*)	*x* (*x*)	*x* (*x*)	*x* (*x*)	*x* (*x*)
**Received ANC in a public sector facility**								
No	*x* (*x*)	*x* (*x*)	*x* (*x*)	*x* (*x*)	*x* (*x*)	*x* (*x*)	*x* (*x*)	*x* (*x*)
Yes	*x* (*x*)	*x* (*x*)	*x* (*x*)	*x* (*x*)	*x* (*x*)	*x* (*x*)	*x* (*x*)	*x* (*x*)
Do not know	*x* (*x*)	*x* (*x*)	*x* (*x*)	*x* (*x*)	*x* (*x*)	*x* (*x*)	*x* (*x*)	*x* (*x*)
**Reasons for not receiving more antenatal care**								
Someone did not allow me to go	*x* (*x*)	*x* (*x*)	*x* (*x*)	*x* (*x*)	*x* (*x*)	*x* (*x*)	*x* (*x*)	*x* (*x*)
I did not know I could go	*x* (*x*)	*x* (*x*)	*x* (*x*)	*x* (*x*)	*x* (*x*)	*x* (*x*)	*x* (*x*)	*x* (*x*)
I did not have money	*x* (*x*)	*x* (*x*)	*x* (*x*)	*x* (*x*)	*x* (*x*)	*x* (*x*)	*x* (*x*)	*x* (*x*)
I did not have time	*x* (*x*)	*x* (*x*)	*x* (*x*)	*x* (*x*)	*x* (*x*)	*x* (*x*)	*x* (*x*)	*x* (*x*)
Health facility was too far	*x* (*x*)	*x* (*x*)	*x* (*x*)	*x* (*x*)	*x* (*x*)	*x* (*x*)	*x* (*x*)	*x* (*x*)
I did not think ANC was necessary for me	*x* (*x*)	*x* (*x*)	*x* (*x*)	*x* (*x*)	*x* (*x*)	*x* (*x*)	*x* (*x*)	*x* (*x*)
I delivered before my last ANC	*x* (*x*)	*x* (*x*)	*x* (*x*)	*x* (*x*)	*x* (*x*)	*x* (*x*)	*x* (*x*)	*x* (*x*)
I was not in the village	*x* (*x*)	*x* (*x*)	*x* (*x*)	*x* (*x*)	*x* (*x*)	*x* (*x*)	*x* (*x*)	*x* (*x*)
Doctor/ANM was not available	*x* (*x*)	*x* (*x*)	*x* (*x*)	*x* (*x*)	*x* (*x*)	*x* (*x*)	*x* (*x*)	*x* (*x*)
I was not feeling well	*x* (*x*)	*x* (*x*)	*x* (*x*)	*x* (*x*)	*x* (*x*)	*x* (*x*)	*x* (*x*)	*x* (*x*)
I did not have someone to go with or help me with my children/other person/duty	*x* (*x*)	*x* (*x*)	*x* (*x*)	*x* (*x*)	*x* (*x*)	*x* (*x*)	*x* (*x*)	*x* (*x*)
I was afraid of COVID19	*x* (*x*)	*x* (*x*)	*x* (*x*)	*x* (*x*)	*x* (*x*)	*x* (*x*)	*x* (*x*)	*x* (*x*)
Others	*x* (*x*)	*x* (*x*)	*x* (*x*)	*x* (*x*)	*x* (*x*)	*x* (*x*)	*x* (*x*)	*x* (*x*)
Do not know	*x* (*x*)	*x* (*x*)	*x* (*x*)	*x* (*x*)	*x* (*x*)	*x* (*x*)	*x* (*x*)	*x* (*x*)
**Why did not you receive antenatal care earlier in the pregnancy?**								
Someone did not allow me to go	*x* (*x*)	*x* (*x*)	*x* (*x*)	*x* (*x*)	*x* (*x*)	*x* (*x*)	*x* (*x*)	*x* (*x*)
I did not know I could go	*x* (*x*)	*x* (*x*)	*x* (*x*)	*x* (*x*)	*x* (*x*)	*x* (*x*)	*x* (*x*)	*x* (*x*)
I did not have money	*x* (*x*)	*x* (*x*)	*x* (*x*)	*x* (*x*)	*x* (*x*)	*x* (*x*)	*x* (*x*)	*x* (*x*)
I did not have time	*x* (*x*)	*x* (*x*)	*x* (*x*)	*x* (*x*)	*x* (*x*)	*x* (*x*)	*x* (*x*)	*x* (*x*)
Health facility was too far	*x* (*x*)	*x* (*x*)	*x* (*x*)	*x* (*x*)	*x* (*x*)	*x* (*x*)	*x* (*x*)	*x* (*x*)
I did not think ANC was necessary for me	*x* (*x*)	*x* (*x*)	*x* (*x*)	*x* (*x*)	*x* (*x*)	*x* (*x*)	*x* (*x*)	*x* (*x*)
I was not in the village	*x* (*x*)	*x* (*x*)	*x* (*x*)	*x* (*x*)	*x* (*x*)	*x* (*x*)	*x* (*x*)	*x* (*x*)
Doctor/ANM was not available	*x* (*x*)	*x* (*x*)	*x* (*x*)	*x* (*x*)	*x* (*x*)	*x* (*x*)	*x* (*x*)	*x* (*x*)
I was not feeling well	*x* (*x*)	*x* (*x*)	*x* (*x*)	*x* (*x*)	*x* (*x*)	*x* (*x*)	*x* (*x*)	*x* (*x*)
I did not have someone to go with or help me with my children/other person/duty	*x* (*x*)	*x* (*x*)	*x* (*x*)	*x* (*x*)	*x* (*x*)	*x* (*x*)	*x* (*x*)	*x* (*x*)
I was afraid of COVID19	*x* (*x*)	*x* (*x*)	*x* (*x*)	*x* (*x*)	*x* (*x*)	*x* (*x*)	*x* (*x*)	*x* (*x*)
Others	*x* (*x*)	*x* (*x*)	*x* (*x*)	*x* (*x*)	*x* (*x*)	*x* (*x*)	*x* (*x*)	*x* (*x*)
**Why did you not take IFA tablets for at least 100 days?**								
I was not told to take IFA for at least 100 days	*x* (*x*)	*x* (*x*)	*x* (*x*)	*x* (*x*)	*x* (*x*)	*x* (*x*)	*x* (*x*)	*x* (*x*)
I was not given IFA for 100 days	*x* (*x*)	*x* (*x*)	*x* (*x*)	*x* (*x*)	*x* (*x*)	*x* (*x*)	*x* (*x*)	*x* (*x*)
I was not feeling well when I started taking the IFA tablets (constipation, vomiting, diarrhoea, black stools)	*x* (*x*)	*x* (*x*)	*x* (*x*)	*x* (*x*)	*x* (*x*)	*x* (*x*)	*x* (*x*)	*x* (*x*)
I received them but forgot to take them	*x* (*x*)	*x* (*x*)	*x* (*x*)	*x* (*x*)	*x* (*x*)	*x* (*x*)	*x* (*x*)	*x* (*x*)
I did not feel IFA intake was necessary	*x* (*x*)	*x* (*x*)	*x* (*x*)	*x* (*x*)	*x* (*x*)	*x* (*x*)	*x* (*x*)	*x* (*x*)
My family member told me not to take IFA	*x* (*x*)	*x* (*x*)	*x* (*x*)	*x* (*x*)	*x* (*x*)	*x* (*x*)	*x* (*x*)	*x* (*x*)
Others	*x* (*x*)	*x* (*x*)	*x* (*x*)	*x* (*x*)	*x* (*x*)	*x* (*x*)	*x* (*x*)	*x* (*x*)
**Did you see anyone for antenatal care during the pregnancy?**								
No	*x* (*x*)	*x* (*x*)	*x* (*x*)	*x* (*x*)	*x* (*x*)	*x* (*x*)	*x* (*x*)	*x* (*x*)
Yes	*x* (*x*)	*x* (*x*)	*x* (*x*)	*x* (*x*)	*x* (*x*)	*x* (*x*)	*x* (*x*)	*x* (*x*)
Missing	*x* (*x*)	*x* (*x*)	*x* (*x*)	*x* (*x*)	*x* (*x*)	*x* (*x*)	*x* (*x*)	*x* (*x*)
**Where did you receive antenatal care during the pregnancy?** ^**d**^								
Sub centre	*x* (*x*)	*x* (*x*)	*x* (*x*)	*x* (*x*)	*x* (*x*)	*x* (*x*)	*x* (*x*)	*x* (*x*)
Primary health centre	*x* (*x*)	*x* (*x*)	*x* (*x*)	*x* (*x*)	*x* (*x*)	*x* (*x*)	*x* (*x*)	*x* (*x*)
Community health centre	*x* (*x*)	*x* (*x*)	*x* (*x*)	*x* (*x*)	*x* (*x*)	*x* (*x*)	*x* (*x*)	*x* (*x*)
District hospital	*x* (*x*)	*x* (*x*)	*x* (*x*)	*x* (*x*)	*x* (*x*)	*x* (*x*)	*x* (*x*)	*x* (*x*)
Civil hospital	*x* (*x*)	*x* (*x*)	*x* (*x*)	*x* (*x*)	*x* (*x*)	*x* (*x*)	*x* (*x*)	*x* (*x*)
Private hospital/clinic	*x* (*x*)	*x* (*x*)	*x* (*x*)	*x* (*x*)	*x* (*x*)	*x* (*x*)	*x* (*x*)	*x* (*x*)
NGO facility	*x* (*x*)	*x* (*x*)	*x* (*x*)	*x* (*x*)	*x* (*x*)	*x* (*x*)	*x* (*x*)	*x* (*x*)
Anganwadi centre	*x* (*x*)	*x* (*x*)	*x* (*x*)	*x* (*x*)	*x* (*x*)	*x* (*x*)	*x* (*x*)	*x* (*x*)
In the village by a mobile team	*x* (*x*)	*x* (*x*)	*x* (*x*)	*x* (*x*)	*x* (*x*)	*x* (*x*)	*x* (*x*)	*x* (*x*)
At home	*x* (*x*)	*x* (*x*)	*x* (*x*)	*x* (*x*)	*x* (*x*)	*x* (*x*)	*x* (*x*)	*x* (*x*)
Vaccination point in the village	*x* (*x*)	*x* (*x*)	*x* (*x*)	*x* (*x*)	*x* (*x*)	*x* (*x*)	*x* (*x*)	*x* (*x*)
Other	*x* (*x*)	*x* (*x*)	*x* (*x*)	*x* (*x*)	*x* (*x*)	*x* (*x*)	*x* (*x*)	*x* (*x*)
Do not know	*x* (*x*)	*x* (*x*)	*x* (*x*)	*x* (*x*)	*x* (*x*)	*x* (*x*)	*x* (*x*)	*x* (*x*)
Missing	*x* (*x*)	*x* (*x*)	*x* (*x*)	*x* (*x*)	*x* (*x*)	*x* (*x*)	*x* (*x*)	*x* (*x*)
**Who did you see for antenatal care during the pregnancy?**								
Doctor	*x* (*x*)	*x* (*x*)	*x* (*x*)	*x* (*x*)	*x* (*x*)	*x* (*x*)	*x* (*x*)	*x* (*x*)
Auxiliary nurse midwife	*x* (*x*)	*x* (*x*)	*x* (*x*)	*x* (*x*)	*x* (*x*)	*x* (*x*)	*x* (*x*)	*x* (*x*)
ASHA	*x* (*x*)	*x* (*x*)	*x* (*x*)	*x* (*x*)	*x* (*x*)	*x* (*x*)	*x* (*x*)	*x* (*x*)
Traditional birth attendant	*x* (*x*)	*x* (*x*)	*x* (*x*)	*x* (*x*)	*x* (*x*)	*x* (*x*)	*x* (*x*)	*x* (*x*)
Anganwadi worker	*x* (*x*)	*x* (*x*)	*x* (*x*)	*x* (*x*)	*x* (*x*)	*x* (*x*)	*x* (*x*)	*x* (*x*)
Registered medical practitioner (RMP)	*x* (*x*)	*x* (*x*)	*x* (*x*)	*x* (*x*)	*x* (*x*)	*x* (*x*)	*x* (*x*)	*x* (*x*)
Bengali doctor	*x* (*x*)	*x* (*x*)	*x* (*x*)	*x* (*x*)	*x* (*x*)	*x* (*x*)	*x* (*x*)	*x* (*x*)
Do not know/do not remember	*x* (*x*)	*x* (*x*)	*x* (*x*)	*x* (*x*)	*x* (*x*)	*x* (*x*)	*x* (*x*)	*x* (*x*)
Missing	*x* (*x*)	*x* (*x*)	*x* (*x*)	*x* (*x*)	*x* (*x*)	*x* (*x*)	*x* (*x*)	*x* (*x*)
**During ANC contacts, was height measured?**								
No	*x* (*x*)	*x* (*x*)	*x* (*x*)	*x* (*x*)	*x* (*x*)	*x* (*x*)	*x* (*x*)	*x* (*x*)
Yes	*x* (*x*)	*x* (*x*)	*x* (*x*)	*x* (*x*)	*x* (*x*)	*x* (*x*)	*x* (*x*)	*x* (*x*)
Missing	*x* (*x*)	*x* (*x*)	*x* (*x*)	*x* (*x*)	*x* (*x*)	*x* (*x*)	*x* (*x*)	*x* (*x*)
**During ANC contacts, was weight measured at least once?**								
No	*x* (*x*)	*x* (*x*)	*x* (*x*)	*x* (*x*)	*x* (*x*)	*x* (*x*)	*x* (*x*)	*x* (*x*)
Yes	*x* (*x*)	*x* (*x*)	*x* (*x*)	*x* (*x*)	*x* (*x*)	*x* (*x*)	*x* (*x*)	*x* (*x*)
Missing	*x* (*x*)	*x* (*x*)	*x* (*x*)	*x* (*x*)	*x* (*x*)	*x* (*x*)	*x* (*x*)	*x* (*x*)
**During ANC contacts, was blood pressure measured at least once?**								
No	*x* (*x*)	*x* (*x*)	*x* (*x*)	*x* (*x*)	*x* (*x*)	*x* (*x*)	*x* (*x*)	*x* (*x*)
Yes	*x* (*x*)	*x* (*x*)	*x* (*x*)	*x* (*x*)	*x* (*x*)	*x* (*x*)	*x* (*x*)	*x* (*x*)
Missing	*x* (*x*)	*x* (*x*)	*x* (*x*)	*x* (*x*)	*x* (*x*)	*x* (*x*)	*x* (*x*)	*x* (*x*)
**During ANC contacts, was there an abdominal examination without a machine?**								
No	*x* (*x*)	*x* (*x*)	*x* (*x*)	*x* (*x*)	*x* (*x*)	*x* (*x*)	*x* (*x*)	*x* (*x*)
Yes	*x* (*x*)	*x* (*x*)	*x* (*x*)	*x* (*x*)	*x* (*x*)	*x* (*x*)	*x* (*x*)	*x* (*x*)
Missing	*x* (*x*)	*x* (*x*)	*x* (*x*)	*x* (*x*)	*x* (*x*)	*x* (*x*)	*x* (*x*)	*x* (*x*)
**During ANC contacts, was there an abdominal examination with a machine?**								
No	*x* (*x*)	*x* (*x*)	*x* (*x*)	*x* (*x*)	*x* (*x*)	*x* (*x*)	*x* (*x*)	*x* (*x*)
Yes	*x* (*x*)	*x* (*x*)	*x* (*x*)	*x* (*x*)	*x* (*x*)	*x* (*x*)	*x* (*x*)	*x* (*x*)
Missing	*x* (*x*)	*x* (*x*)	*x* (*x*)	*x* (*x*)	*x* (*x*)	*x* (*x*)	*x* (*x*)	*x* (*x*)
**During ANC contacts, was there an abdominal examination with or without a machine?**								
No	*x* (*x*)	*x* (*x*)	*x* (*x*)	*x* (*x*)	*x* (*x*)	*x* (*x*)	*x* (*x*)	*x* (*x*)
Yes	*x* (*x*)	*x* (*x*)	*x* (*x*)	*x* (*x*)	*x* (*x*)	*x* (*x*)	*x* (*x*)	*x* (*x*)
Missing	*x* (*x*)	*x* (*x*)	*x* (*x*)	*x* (*x*)	*x* (*x*)	*x* (*x*)	*x* (*x*)	*x* (*x*)
**During ANC contacts, was tetanus vaccination given?**								
No	*x* (*x*)	*x* (*x*)	*x* (*x*)	*x* (*x*)	*x* (*x*)	*x* (*x*)	*x* (*x*)	*x* (*x*)
Yes	*x* (*x*)	*x* (*x*)	*x* (*x*)	*x* (*x*)	*x* (*x*)	*x* (*x*)	*x* (*x*)	*x* (*x*)
Missing	*x* (*x*)	*x* (*x*)	*x* (*x*)	*x* (*x*)	*x* (*x*)	*x* (*x*)	*x* (*x*)	*x* (*x*)
**During ANC contacts, was there a urine test?**								
No	*x* (*x*)	*x* (*x*)	*x* (*x*)	*x* (*x*)	*x* (*x*)	*x* (*x*)	*x* (*x*)	*x* (*x*)
Yes	*x* (*x*)	*x* (*x*)	*x* (*x*)	*x* (*x*)	*x* (*x*)	*x* (*x*)	*x* (*x*)	*x* (*x*)
Missing	*x* (*x*)	*x* (*x*)	*x* (*x*)	*x* (*x*)	*x* (*x*)	*x* (*x*)	*x* (*x*)	*x* (*x*)
**During ANC contacts, was there a blood test?**								
No	*x* (*x*)	*x* (*x*)	*x* (*x*)	*x* (*x*)	*x* (*x*)	*x* (*x*)	*x* (*x*)	*x* (*x*)
Yes	*x* (*x*)	*x* (*x*)	*x* (*x*)	*x* (*x*)	*x* (*x*)	*x* (*x*)	*x* (*x*)	*x* (*x*)
Missing	*x* (*x*)	*x* (*x*)	*x* (*x*)	*x* (*x*)	*x* (*x*)	*x* (*x*)	*x* (*x*)	*x* (*x*)
**During ANC contacts, was there counselling on healthy eating?**								
No	*x* (*x*)	*x* (*x*)	*x* (*x*)	*x* (*x*)	*x* (*x*)	*x* (*x*)	*x* (*x*)	*x* (*x*)
Yes	*x* (*x*)	*x* (*x*)	*x* (*x*)	*x* (*x*)	*x* (*x*)	*x* (*x*)	*x* (*x*)	*x* (*x*)
Missing	*x* (*x*)	*x* (*x*)	*x* (*x*)	*x* (*x*)	*x* (*x*)	*x* (*x*)	*x* (*x*)	*x* (*x*)
**During ANC contacts, was there counselling on personal hygiene?**								
No	*x* (*x*)	*x* (*x*)	*x* (*x*)	*x* (*x*)	*x* (*x*)	*x* (*x*)	*x* (*x*)	*x* (*x*)
Yes	*x* (*x*)	*x* (*x*)	*x* (*x*)	*x* (*x*)	*x* (*x*)	*x* (*x*)	*x* (*x*)	*x* (*x*)
Missing	*x* (*x*)	*x* (*x*)	*x* (*x*)	*x* (*x*)	*x* (*x*)	*x* (*x*)	*x* (*x*)	*x* (*x*)
**During ANC contacts, was there counselling on adequate sleep and rest during the day?**								
No	*x* (*x*)	*x* (*x*)	*x* (*x*)	*x* (*x*)	*x* (*x*)	*x* (*x*)	*x* (*x*)	*x* (*x*)
Yes	*x* (*x*)	*x* (*x*)	*x* (*x*)	*x* (*x*)	*x* (*x*)	*x* (*x*)	*x* (*x*)	*x* (*x*)
Missing	*x* (*x*)	*x* (*x*)	*x* (*x*)	*x* (*x*)	*x* (*x*)	*x* (*x*)	*x* (*x*)	*x* (*x*)
**During ANC contacts, was there counselling on avoiding strenuous activity/lifting heavy weights?**								
No	*x* (*x*)	*x* (*x*)	*x* (*x*)	*x* (*x*)	*x* (*x*)	*x* (*x*)	*x* (*x*)	*x* (*x*)
Yes	*x* (*x*)	*x* (*x*)	*x* (*x*)	*x* (*x*)	*x* (*x*)	*x* (*x*)	*x* (*x*)	*x* (*x*)
Missing	*x* (*x*)	*x* (*x*)	*x* (*x*)	*x* (*x*)	*x* (*x*)	*x* (*x*)	*x* (*x*)	*x* (*x*)
**During ANC contacts, was there counselling about not smoking/drinking alcohol?**								
No	*x* (*x*)	*x* (*x*)	*x* (*x*)	*x* (*x*)	*x* (*x*)	*x* (*x*)	*x* (*x*)	*x* (*x*)
Yes	*x* (*x*)	*x* (*x*)	*x* (*x*)	*x* (*x*)	*x* (*x*)	*x* (*x*)	*x* (*x*)	*x* (*x*)
Missing	*x* (*x*)	*x* (*x*)	*x* (*x*)	*x* (*x*)	*x* (*x*)	*x* (*x*)	*x* (*x*)	*x* (*x*)
**During ANC contacts, was there counselling about all five of healthy eating, personal hygiene, adequate sleep and rest during the day, not smoking/drinking alcohol, and avoiding strenuous activity/lifting heavy weights?**								
No	*x* (*x*)	*x* (*x*)	*x* (*x*)	*x* (*x*)	*x* (*x*)	*x* ( *x*)	*x* ( *x*)	*x* ( *x*)
Yes	*x* (*x*)	*x* (*x*)	*x* (*x*)	*x* (*x*)	*x* ( *x*)	*x* ( *x*)	*x* ( *x*)	*x* ( *x*)
Missing	*x* (*x*)	*x* (*x*)	*x* (*x*)	*x* (*x*)	*x* ( *x*)	*x* ( *x*)	*x* ( *x*)	*x* ( *x*)
**Other ANC advice**								
*x*	*x* ( *x*)	*x* ( *x*)	*x* (*x*)	*x* (*x*)	*x* ( *x*)	*x* ( *x*)	*x* ( *x*)	*x* ( *x*)
*y*	*x* ( *x*)	*x* ( *x*)	*x* (*x*)	*x* (*x*)	*x* ( *x*)	*x* ( *x*)	*x* ( *x*)	*x* ( *x*)
*z*	*x* ( *x*)	*x* ( *x*)	*x* (*x*)	*x* (*x*)	*x* ( *x*)	*x* ( *x*)	*x* ( *x*)	*x* ( *x*)
**Number of procedures followed** ^**e**^								
0	*x* ( *x*)	*x* ( *x*)	*x* (*x*)	*x* (*x*)	*x* ( *x*)	*x* ( *x*)	*x* ( *x*)	*x* ( *x*)
1	*x* ( *x*)	*x* ( *x*)	*x* (*x*)	*x* (*x*)	*x* ( *x*)	*x* ( *x*)	*x* ( *x*)	*x* ( *x*)
2	*x* ( *x*)	*x* ( *x*)	*x* (*x*)	*x* (*x*)	*x* ( *x*)	*x* ( *x*)	*x* ( *x*)	*x* ( *x*)
3	*x* ( *x*)	*x* ( *x*)	*x* (*x*)	*x* (*x*)	*x* ( *x*)	*x* ( *x*)	*x* ( *x*)	*x* ( *x*)
4	*x* ( *x*)	*x* ( *x*)	*x* (*x*)	*x* (*x*)	*x* ( *x*)	*x* ( *x*)	*x* ( *x*)	*x* ( *x*)
5	*x* ( *x*)	*x* ( *x*)	*x* (*x*)	*x* (*x*)	*x* ( *x*)	*x* ( *x*)	*x* ( *x*)	*x* ( *x*)
6	*x* ( *x*)	*x* ( *x*)	*x* (*x*)	*x* (*x*)	*x* ( *x*)	*x* ( *x*)	*x* ( *x*)	*x* ( *x*)
7	*x* ( *x*)	*x* ( *x*)	*x* (*x*)	*x* (*x*)	*x* ( *x*)	*x* ( *x*)	*x* ( *x*)	*x* ( *x*)
8	*x* ( *x*)	*x* ( *x*)	*x* (*x*)	*x* (*x*)	*x* ( *x*)	*x* ( *x*)	*x* ( *x*)	*x* ( *x*)
**Postnatal care**								
**Did any nurse/doctor or any other health worker check on your health in the first 2 days after delivery?**								
Yes	*x* ( *x*)	*x* ( *x*)	*x* (*x*)	*x* (*x*)	*x* ( *x*)	*x* ( *x*)	*x* ( *x*)	*x* ( *x*)
No	*x* ( *x*)	*x* ( *x*)	*x* (*x*)	*x* (*x*)	*x* ( *x*)	*x* ( *x*)	*x* ( *x*)	*x* ( *x*)
Missing	*x* ( *x*)	*x* ( *x*)	*x* (*x*)	*x* (*x*)	*x* ( *x*)	*x* ( *x*)	*x* ( *x*)	*x* ( *x*)
**Who checked on your health in the first 2 days after delivery?**								
Doctor	*x* ( *x*)	*x* ( *x*)	*x* (*x*)	*x* (*x*)	*x* ( *x*)	*x* ( *x*)	*x* ( *x*)	*x* ( *x*)
Auxiliary nurse midwife	*x* ( *x*)	*x* ( *x*)	*x* (*x*)	*x* (*x*)	*x* ( *x*)	*x* ( *x*)	*x* ( *x*)	*x* ( *x*)
ASHA	*x* ( *x*)	*x* ( *x*)	*x* (*x*)	*x* (*x*)	*x* ( *x*)	*x* ( *x*)	*x* ( *x*)	*x* ( *x*)
Traditional birth attendant	*x* ( *x*)	*x* ( *x*)	*x* (*x*)	*x* (*x*)	*x* ( *x*)	*x* ( *x*)	*x* ( *x*)	*x* ( *x*)
Anganwadi worker	*x* ( *x*)	*x* ( *x*)	*x* (*x*)	*x* (*x*)	*x* ( *x*)	*x* ( *x*)	*x* ( *x*)	*x* ( *x*)
Registered medical practitioner (RMP)	*x* ( *x*)	*x* ( *x*)	*x* (*x*)	*x* (*x*)	*x* ( *x*)	*x* ( *x*)	*x* ( *x*)	*x* ( *x*)
Bengali doctor	*x* ( *x*)	*x* ( *x*)	*x* (*x*)	*x* (*x*)	*x* ( *x*)	*x* ( *x*)	*x* ( *x*)	*x* ( *x*)
Other	*x* ( *x*)	*x* ( *x*)	*x* (*x*)	*x* (*x*)	*x* ( *x*)	*x* ( *x*)	*x* ( *x*)	*x* ( *x*)
Do not know/do not remember	*x* ( *x*)	*x* ( *x*)	*x* (*x*)	*x* (*x*)	*x* ( *x*)	*x* ( *x*)	*x* ( *x*)	*x* ( *x*)
Missing	*x* ( *x*)	*x* ( *x*)	*x* (*x*)	*x* (*x*)	*x* ( *x*)	*x* ( *x*)	*x* ( *x*)	*x* ( *x*)
**During these visits, were you checked/advised about the following?**								
**Checked for fever after delivery**								
Yes	*x* ( *x*)	*x* ( *x*)	*x* (*x*)	*x* (*x*)	*x* ( *x*)	*x* ( *x*)	*x* ( *x*)	*x* ( *x*)
No	*x* ( *x*)	*x* ( *x*)	*x* (*x*)	*x* (*x*)	*x* ( *x*)	*x* ( *x*)	*x* ( *x*)	*x* ( *x*)
Do not know	*x* ( *x*)	*x* ( *x*)	*x* (*x*)	*x* (*x*)	*x* ( *x*)	*x* ( *x*)	*x* ( *x*)	*x* ( *x*)
Missing	*x* ( *x*)	*x* ( *x*)	*x* (*x*)	*x* (*x*)	*x* ( *x*)	*x* ( *x*)	*x* ( *x*)	*x* ( *x*)
**Examined abdomen**								
Yes	*x* ( *x*)	*x* ( *x*)	*x* (*x*)	*x* (*x*)	*x* ( *x*)	*x* ( *x*)	*x* ( *x*)	*x* ( *x*)
No	*x* ( *x*)	*x* ( *x*)	*x* (*x*)	*x* (*x*)	*x* ( *x*)	*x* ( *x*)	*x* ( *x*)	*x* ( *x*)
Do not know	*x* ( *x*)	*x* ( *x*)	*x* (*x*)	*x* (*x*)	*x* ( *x*)	*x* ( *x*)	*x* ( *x*)	*x* ( *x*)
Missing	*x* ( *x*)	*x* ( *x*)	*x* (*x*)	*x* (*x*)	*x* ( *x*)	*x* ( *x*)	*x* ( *x*)	*x* ( *x*)
**Checked blood pressure**								
Yes	*x* ( *x*)	*x* ( *x*)	*x* (*x*)	*x* (*x*)	*x* ( *x*)	*x* ( *x*)	*x* ( *x*)	*x* ( *x*)
No	*x* ( *x*)	*x* ( *x*)	*x* (*x*)	*x* (*x*)	*x* ( *x*)	*x* ( *x*)	*x* ( *x*)	*x* ( *x*)
Do not know	*x* ( *x*)	*x* ( *x*)	*x* (*x*)	*x* (*x*)	*x* ( *x*)	*x* ( *x*)	*x* ( *x*)	*x* ( *x*)
Missing	*x* ( *x*)	*x* ( *x*)	*x* (*x*)	*x* (*x*)	*x* ( *x*)	*x* ( *x*)	*x* ( *x*)	*x* ( *x*)
**Asked about excessive vaginal bleeding**								
Yes	*x* ( *x*)	*x* ( *x*)	*x* (*x*)	*x* (*x*)	*x* ( *x*)	*x* ( *x*)	*x* ( *x*)	*x* ( *x*)
No	*x* ( *x*)	*x* ( *x*)	*x* (*x*)	*x* (*x*)	*x* ( *x*)	*x* ( *x*)	*x* ( *x*)	*x* ( *x*)
Do not know	*x* ( *x*)	*x* ( *x*)	*x* (*x*)	*x* (*x*)	*x* ( *x*)	*x* ( *x*)	*x* ( *x*)	*x* ( *x*)
Missing	*x* ( *x*)	*x* ( *x*)	*x* (*x*)	*x* (*x*)	*x* ( *x*)	*x* ( *x*)	*x* ( *x*)	*x* ( *x*)
**Asked about fits**								
Yes	*x* ( *x*)	*x* ( *x*)	*x* (*x*)	*x* (*x*)	*x* ( *x*)	*x* ( *x*)	*x* ( *x*)	*x* ( *x*)
No	*x* ( *x*)	*x* ( *x*)	*x* (*x*)	*x* (*x*)	*x* ( *x*)	*x* ( *x*)	*x* ( *x*)	*x* ( *x*)
Do not know	*x* ( *x*)	*x* ( *x*)	*x* (*x*)	*x* (*x*)	*x* ( *x*)	*x* ( *x*)	*x* ( *x*)	*x* ( *x*)
Missing	*x* ( *x*)	*x* ( *x*)	*x* (*x*)	*x* (*x*)	*x* ( *x*)	*x* ( *x*)	*x* ( *x*)	*x* ( *x*)
**Advised about iron tablets (100 IFA)**								
Yes	*x* ( *x*)	*x* ( *x*)	*x* (*x*)	*x* (*x*)	*x* ( *x*)	*x* ( *x*)	*x* ( *x*)	*x* ( *x*)
No	*x* ( *x*)	*x* ( *x*)	*x* (*x*)	*x* (*x*)	*x* ( *x*)	*x* ( *x*)	*x* ( *x*)	*x* ( *x*)
Do not know	*x* ( *x*)	*x* ( *x*)	*x* (*x*)	*x* (*x*)	*x* ( *x*)	*x* ( *x*)	*x* ( *x*)	*x* ( *x*)
Missing	*x* ( *x*)	*x* ( *x*)	*x* (*x*)	*x* (*x*)	*x* ( *x*)	*x* ( *x*)	*x* ( *x*)	*x* ( *x*)
**Advised about nutrition**								
Yes	*x* ( *x*)	*x* ( *x*)	*x* (*x*)	*x* (*x*)	*x* ( *x*)	*x* ( *x*)	*x* ( *x*)	*x* ( *x*)
No	*x* ( *x*)	*x* ( *x*)	*x* (*x*)	*x* (*x*)	*x* ( *x*)	*x* ( *x*)	*x* ( *x*)	*x* ( *x*)
Do not know	*x* ( *x*)	*x* ( *x*)	*x* (*x*)	*x* (*x*)	*x* ( *x*)	*x* ( *x*)	*x* ( *x*)	*x* ( *x*)
Missing	*x* ( *x*)	*x* ( *x*)	*x* (*x*)	*x* (*x*)	*x* ( *x*)	*x* ( *x*)	*x* ( *x*)	*x* ( *x*)
**Advised about exclusive breastfeeding**								
Yes	*x* ( *x*)	*x* ( *x*)	*x* (*x*)	*x* (*x*)	*x* ( *x*)	*x* ( *x*)	*x* ( *x*)	*x* ( *x*)
No	*x* ( *x*)	*x* ( *x*)	*x* (*x*)	*x* (*x*)	*x* ( *x*)	*x* ( *x*)	*x* ( *x*)	*x* ( *x*)
Do not know	*x* ( *x*)	*x* ( *x*)	*x* (*x*)	*x* (*x*)	*x* ( *x*)	*x* ( *x*)	*x* ( *x*)	*x* ( *x*)
Missing	*x* ( *x*)	*x* ( *x*)	*x* (*x*)	*x* (*x*)	*x* ( *x*)	*x* ( *x*)	*x* ( *x*)	*x* ( *x*)
**Advised about family planning**								
Yes	*x* ( *x*)	*x* ( *x*)	*x* (*x*)	*x* (*x*)	*x* ( *x*)	*x* ( *x*)	*x* ( *x*)	*x* ( *x*)
No	*x* ( *x*)	*x* ( *x*)	*x* (*x*)	*x* (*x*)	*x* ( *x*)	*x* ( *x*)	*x* ( *x*)	*x* ( *x*)
Do not know	*x* ( *x*)	*x* ( *x*)	*x* (*x*)	*x* (*x*)	*x* ( *x*)	*x* ( *x*)	*x* ( *x*)	*x* ( *x*)
Missing	*x* ( *x*)	*x* ( *x*)	*x* (*x*)	*x* (*x*)	*x* ( *x*)	*x* ( *x*)	*x* ( *x*)	*x* ( *x*)
**Advised about nutrition, exclusive breastfeeding and family planning**								
Yes	*x* ( *x*)	*x* ( *x*)	*x* (*x*)	*x* (*x*)	*x* ( *x*)	*x* ( *x*)	*x* ( *x*)	*x* ( *x*)
No	*x* ( *x*)	*x* ( *x*)	*x* (*x*)	*x* (*x*)	*x* ( *x*)	*x* ( *x*)	*x* ( *x*)	*x* ( *x*)
Do not know	*x* ( *x*)	*x* ( *x*)	*x* (*x*)	*x* (*x*)	*x* ( *x*)	*x* ( *x*)	*x* ( *x*)	*x* ( *x*)
Missing	*x* ( *x*)	*x* ( *x*)	*x* (*x*)	*x* (*x*)	*x* ( *x*)	*x* ( *x*)	*x* ( *x*)	*x* ( *x*)
**Number of procedures followed** ^**f**^								
0	*x* ( *x*)	*x* ( *x*)	*x* (*x*)	*x* (*x*)	*x* ( *x*)	*x* ( *x*)	*x* ( *x*)	*x* ( *x*)
1	*x* ( *x*)	*x* ( *x*)	*x* (*x*)	*x* (*x*)	*x* ( *x*)	*x* ( *x*)	*x* ( *x*)	*x* ( *x*)
2	*x* ( *x*)	*x* ( *x*)	*x* (*x*)	*x* (*x*)	*x* ( *x*)	*x* ( *x*)	*x* ( *x*)	*x* ( *x*)
3	*x* ( *x*)	*x* ( *x*)	*x* (*x*)	*x* (*x*)	*x* ( *x*)	*x* ( *x*)	*x* ( *x*)	*x* ( *x*)
4	*x* ( *x*)	*x* ( *x*)	*x* (*x*)	*x* (*x*)	*x* ( *x*)	*x* ( *x*)	*x* ( *x*)	*x* ( *x*)
5	*x* ( *x*)	*x* ( *x*)	*x* (*x*)	*x* (*x*)	*x* ( *x*)	*x* ( *x*)	*x* ( *x*)	*x* ( *x*)
6	*x* ( *x*)	*x* ( *x*)	*x* (*x*)	*x* (*x*)	*x* ( *x*)	*x* ( *x*)	*x* ( *x*)	*x* ( *x*)
7	*x* ( *x*)	*x* ( *x*)	*x* (*x*)	*x* (*x*)	*x* ( *x*)	*x* ( *x*)	*x* ( *x*)	*x* ( *x*)

^a^Information was combined using a weighted average (with information from mothers of singleton babies who survived the neonatal period given ten times the weight of others)

^b^IFA tablets for 100 days or equivalent: woman took IFA tablets or other medication (which had iron) or a combination of these for at least 100 days or iron syrup or iron injection/infusion

^c^Full antenatal care: woman had at least four antenatal visits, at least one tetanus toxoid (TT) injection, and took IFA tablets for 100 days or equivalent

^d^Multiple responses are possible

^e^Number of procedures is the sum of the following: weight measurement; blood pressure measurement; urine testing; abdominal examination (with or without a machine); tetanus vaccination; blood test (to assess anaemia and possibly parasite and other infections); prescribed/given and consumed IFA tablets for 100 days or equivalent; counselling on healthy eating, personal hygiene, adequate sleep and rest during the day, and not smoking/drinking alcohol

^f^Number of procedures is the sum of the following: asked about excessive vaginal bleeding; asked about fits; checked for fever after delivery; examined abdomen; checked blood pressure; advised about 100 days of IFA intake; counselled on nutrition, exclusive breastfeeding and family planning

**Table 11:** Delivery, immediate newborn care, and newborn care during baby’s first month (C1) (number (%) unless otherwise stated)

	Singleton babies who were alive at 28 days		Multiple birth babies who were alive at 28 days		Babies who were dead at 28 days		Weighted average of all babies^a^	
	CHAMPION2 intervention arm	STRIPES2 intervention arm	CHAMPION2 intervention arm	STRIPES2 intervention arm	CHAMPION2 intervention arm	STRIPES2 intervention arm	CHAMPION2 intervention arm	STRIPES2 intervention arm
**Intra-natal services received**								
**Place of delivery**								
Hospital (health facility)	*x* ( *x*)	*x* ( *x*)	*x* ( *x*)	*x* ( *x*)	*x* ( *x*)	*x* ( *x*)	*x* ( *x*)	*x* ( *x*)
On the way to hospital (health facility)	*x* ( *x*)	*x* ( *x*)	*x* ( *x*)	*x* ( *x*)	*x* ( *x*)	*x* ( *x*)	*x* ( *x*)	*x* ( *x*)
At home	*x* ( *x*)	*x* ( *x*)	*x* ( *x*)	*x* ( *x*)	*x* ( *x*)	*x* ( *x*)	*x* ( *x*)	*x* ( *x*)
Parent’s home	*x* ( *x*)	*x* ( *x*)	*x* ( *x*)	*x* ( *x*)	*x* ( *x*)	*x* ( *x*)	*x* ( *x*)	*x* ( *x*)
Relative’s home	*x* ( *x*)	*x* ( *x*)	*x* ( *x*)	*x* ( *x*)	*x* ( *x*)	*x* ( *x*)	*x* ( *x*)	*x* ( *x*)
Mother in law’s home	*x* ( *x*)	*x* ( *x*)	*x* ( *x*)	*x* ( *x*)	*x* ( *x*)	*x* ( *x*)	*x* ( *x*)	*x* ( *x*)
Missing	*x* ( *x*)	*x* ( *x*)	*x* ( *x*)	*x* ( *x*)	*x* ( *x*)	*x* ( *x*)	*x* ( *x*)	*x* ( *x*)
**Type of delivery**								
Vaginal (normal/assisted)	*x* ( *x*)	*x* ( *x*)	*x* ( *x*)	*x* ( *x*)	*x* ( *x*)	*x* ( *x*)	*x* ( *x*)	*x* ( *x*)
C-section	*x* ( *x*)	*x* ( *x*)	*x* ( *x*)	*x* ( *x*)	*x* ( *x*)	*x* ( *x*)	*x* ( *x*)	*x* ( *x*)
**Type of hospital (health facility) where you delivered**								
Sub centre	*x* ( *x*)	*x* ( *x*)	*x* ( *x*)	*x* ( *x*)	*x* ( *x*)	*x* ( *x*)	*x* ( *x*)	*x* ( *x*)
Primary health centre	*x* ( *x*)	*x* ( *x*)	*x* ( *x*)	*x* ( *x*)	*x* ( *x*)	*x* ( *x*)	*x* ( *x*)	*x* ( *x*)
Community health centre	*x* ( *x*)	*x* ( *x*)	*x* ( *x*)	*x* ( *x*)	*x* ( *x*)	*x* ( *x*)	*x* ( *x*)	*x* ( *x*)
District hospital	*x* ( *x*)	*x* ( *x*)	*x* ( *x*)	*x* ( *x*)	*x* ( *x*)	*x* ( *x*)	*x* ( *x*)	*x* ( *x*)
Civil hospital	*x* ( *x*)	*x* ( *x*)	*x* ( *x*)	*x* ( *x*)	*x* ( *x*)	*x* ( *x*)	*x* ( *x*)	*x* ( *x*)
Private hospital/clinic	*x* ( *x*)	*x* ( *x*)	*x* ( *x*)	*x* ( *x*)	*x* ( *x*)	*x* ( *x*)	*x* ( *x*)	*x* ( *x*)
NGO facility	*x* ( *x*)	*x* ( *x*)	*x* ( *x*)	*x* ( *x*)	*x* ( *x*)	*x* ( *x*)	*x* ( *x*)	*x* ( *x*)
Others	*x* ( *x*)	*x* ( *x*)	*x* ( *x*)	*x* ( *x*)	*x* ( *x*)	*x* ( *x*)	*x* ( *x*)	*x* ( *x*)
Missing	*x* ( *x*)	*x* ( *x*)	*x* ( *x*)	*x* ( *x*)	*x* ( *x*)	*x* ( *x*)	*x* ( *x*)	*x* ( *x*)
**Safe delivery kit used for non-hospital delivery**								
No	*x* ( *x*)	*x* ( *x*)	*x* ( *x*)	*x* ( *x*)	*x* ( *x*)	*x* ( *x*)	*x* ( *x*)	*x* ( *x*)
Yes	*x* ( *x*)	*x* ( *x*)	*x* ( *x*)	*x* ( *x*)	*x* ( *x*)	*x* ( *x*)	*x* ( *x*)	*x* ( *x*)
Do not know	*x* ( *x*)	*x* ( *x*)	*x* ( *x*)	*x* ( *x*)	*x* ( *x*)	*x* ( *x*)	*x* ( *x*)	*x* ( *x*)
Missing	*x* ( *x*)	*x* ( *x*)	*x* ( *x*)	*x* ( *x*)	*x* ( *x*)	*x* ( *x*)	*x* ( *x*)	*x* ( *x*)
**Person who conducted the delivery**								
Doctor	*x* ( *x*)	*x* ( *x*)	*x* ( *x*)	*x* ( *x*)	*x* ( *x*)	*x* ( *x*)	*x* ( *x*)	*x* ( *x*)
Auxiliary nurse midwife	*x* ( *x*)	*x* ( *x*)	*x* ( *x*)	*x* ( *x*)	*x* ( *x*)	*x* ( *x*)	*x* ( *x*)	*x* ( *x*)
ASHA	*x* ( *x*)	*x* ( *x*)	*x* ( *x*)	*x* ( *x*)	*x* ( *x*)	*x* ( *x*)	*x* ( *x*)	*x* ( *x*)
Traditional birth attendant	*x* ( *x*)	*x* ( *x*)	*x* ( *x*)	*x* ( *x*)	*x* ( *x*)	*x* ( *x*)	*x* ( *x*)	*x* ( *x*)
Anganwadi worker	*x* ( *x*)	*x* ( *x*)	*x* ( *x*)	*x* ( *x*)	*x* ( *x*)	*x* ( *x*)	*x* ( *x*)	*x* ( *x*)
Relative/friend (not trained)	*x* ( *x*)	*x* ( *x*)	*x* ( *x*)	*x* ( *x*)	*x* ( *x*)	*x* ( *x*)	*x* ( *x*)	*x* ( *x*)
Nobody	*x* ( *x*)	*x* ( *x*)	*x* ( *x*)	*x* ( *x*)	*x* ( *x*)	*x* ( *x*)	*x* ( *x*)	*x* ( *x*)
Others	*x* ( *x*)	*x* ( *x*)	*x* ( *x*)	*x* ( *x*)	*x* ( *x*)	*x* ( *x*)	*x* ( *x*)	*x* ( *x*)
Do not know/do not remember	*x* ( *x*)	*x* ( *x*)	*x* ( *x*)	*x* ( *x*)	*x* ( *x*)	*x* ( *x*)	*x* ( *x*)	*x* ( *x*)
**Skilled health professional conducted home delivery**								
No	*x* ( *x*)	*x* ( *x*)	*x* ( *x*)	*x* ( *x*)	*x* ( *x*)	*x* ( *x*)	*x* ( *x*)	*x* ( *x*)
Yes	*x* ( *x*)	*x* ( *x*)	*x* ( *x*)	*x* ( *x*)	*x* ( *x*)	*x* ( *x*)	*x* ( *x*)	*x* ( *x*)
**Reasons for not delivering in hospital**								
Cost too much	*x* ( *x*)	*x* ( *x*)	*x* ( *x*)	*x* ( *x*)	*x* ( *x*)	*x* ( *x*)	*x* ( *x*)	*x* ( *x*)
It was late in the night/hospital not open	*x* ( *x*)	*x* ( *x*)	*x* ( *x*)	*x* ( *x*)	*x* ( *x*)	*x* ( *x*)	*x* ( *x*)	*x* ( *x*)
Hospital was far away/no transport facility available	*x* ( *x*)	*x* ( *x*)	*x* ( *x*)	*x* ( *x*)	*x* ( *x*)	*x* ( *x*)	*x* ( *x*)	*x* ( *x*)
Do not trust/poor quality service	*x* ( *x*)	*x* ( *x*)	*x* ( *x*)	*x* ( *x*)	*x* ( *x*)	*x* ( *x*)	*x* ( *x*)	*x* ( *x*)
No female provider at facility	*x* ( *x*)	*x* ( *x*)	*x* ( *x*)	*x* ( *x*)	*x* ( *x*)	*x* ( *x*)	*x* ( *x*)	*x* ( *x*)
Husband/family did not allow	*x* ( *x*)	*x* ( *x*)	*x* ( *x*)	*x* ( *x*)	*x* ( *x*)	*x* ( *x*)	*x* ( *x*)	*x* ( *x*)
Not necessary	*x* ( *x*)	*x* ( *x*)	*x* ( *x*)	*x* ( *x*)	*x* ( *x*)	*x* ( *x*)	*x* ( *x*)	*x* ( *x*)
Not customary	*x* ( *x*)	*x* ( *x*)	*x* ( *x*)	*x* ( *x*)	*x* ( *x*)	*x* ( *x*)	*x* ( *x*)	*x* ( *x*)
Transportation came late	*x* ( *x*)	*x* ( *x*)	*x* ( *x*)	*x* ( *x*)	*x* ( *x*)	*x* ( *x*)	*x* ( *x*)	*x* ( *x*)
No one to escort me	*x* ( *x*)	*x* ( *x*)	*x* ( *x*)	*x* ( *x*)	*x* ( *x*)	*x* ( *x*)	*x* ( *x*)	*x* ( *x*)
ASHA was not available	*x* ( *x*)	*x* ( *x*)	*x* ( *x*)	*x* ( *x*)	*x* ( *x*)	*x* ( *x*)	*x* ( *x*)	*x* ( *x*)
Too long to decide/delivery was too fast	*x* ( *x*)	*x* ( *x*)	*x* ( *x*)	*x* ( *x*)	*x* ( *x*)	*x* ( *x*)	*x* ( *x*)	*x* ( *x*)
I was afraid of COVID19	*x* ( *x*)	*x* ( *x*)	*x* ( *x*)	*x* ( *x*)	*x* ( *x*)	*x* ( *x*)	*x* ( *x*)	*x* ( *x*)
Other	*x* ( *x*)	*x* ( *x*)	*x* ( *x*)	*x* ( *x*)	*x* ( *x*)	*x* ( *x*)	*x* ( *x*)	*x* ( *x*)
Missing	*x* ( *x*)	*x* ( *x*)	*x* ( *x*)	*x* ( *x*)	*x* ( *x*)	*x* ( *x*)	*x* ( *x*)	*x* ( *x*)
**Immediate care of newborn**								
**Baby placed on your chest with skin-to-skin contact immediately after birth**								
No	*x* ( *x*)	*x* ( *x*)	*x* ( *x*)	*x* ( *x*)	*x* ( *x*)	*x* ( *x*)	*x* ( *x*)	*x* ( *x*)
Yes	*x* ( *x*)	*x* ( *x*)	*x* ( *x*)	*x* ( *x*)	*x* ( *x*)	*x* ( *x*)	*x* ( *x*)	*x* ( *x*)
Do not know	*x* ( *x*)	*x* ( *x*)	*x* ( *x*)	*x* ( *x*)	*x* ( *x*)	*x* ( *x*)	*x* ( *x*)	*x* ( *x*)
Missing	*x* ( *x*)	*x* ( *x*)	*x* ( *x*)	*x* ( *x*)	*x* ( *x*)	*x* ( *x*)	*x* ( *x*)	*x* ( *x*)
**Baby placed on your chest with skin-to-skin contact within 24 h after birth**								
No	*x* ( *x*)	*x* ( *x*)	*x* ( *x*)	*x* ( *x*)	*x* ( *x*)	*x* ( *x*)	*x* ( *x*)	*x* ( *x*)
Yes	*x* ( *x*)	*x* ( *x*)	*x* ( *x*)	*x* ( *x*)	*x* ( *x*)	*x* ( *x*)	*x* ( *x*)	*x* ( *x*)
Missing	*x* ( *x*)	*x* ( *x*)	*x* ( *x*)	*x* ( *x*)	*x* ( *x*)	*x* ( *x*)	*x* ( *x*)	*x* ( *x*)
**Breastfeeding started within 4 h**								
No	*x* ( *x*)	*x* ( *x*)	*x* ( *x*)	*x* ( *x*)	*x* ( *x*)	*x* ( *x*)	*x* ( *x*)	*x* ( *x*)
Yes	*x* ( *x*)	*x* ( *x*)	*x* ( *x*)	*x* ( *x*)	*x* ( *x*)	*x* ( *x*)	*x* ( *x*)	*x* ( *x*)
Missing	*x* ( *x*)	*x* ( *x*)	*x* ( *x*)	*x* ( *x*)	*x* ( *x*)	*x* ( *x*)	*x* ( *x*)	*x* ( *x*)
**Clean cord stump maintained**								
No	*x* ( *x*)	*x* ( *x*)	*x* ( *x*)	*x* ( *x*)	*x* ( *x*)	*x* ( *x*)	*x* ( *x*)	*x* ( *x*)
Yes	*x* ( *x*)	*x* ( *x*)	*x* ( *x*)	*x* ( *x*)	*x* ( *x*)	*x* ( *x*)	*x* ( *x*)	*x* ( *x*)
Do not know	*x* ( *x*)	*x* ( *x*)	*x* ( *x*)	*x* ( *x*)	*x* ( *x*)	*x* ( *x*)	*x* ( *x*)	*x* ( *x*)
Missing	*x* ( *x*)	*x* ( *x*)	*x* ( *x*)	*x* ( *x*)	*x* ( *x*)	*x* ( *x*)	*x* ( *x*)	*x* ( *x*)
**Baby bathed in first 24 h**								
No	*x* ( *x*)	*x* ( *x*)	*x* ( *x*)	*x* ( *x*)	*x* ( *x*)	*x* ( *x*)	*x* ( *x*)	*x* ( *x*)
Yes	*x* ( *x*)	*x* ( *x*)	*x* ( *x*)	*x* ( *x*)	*x* ( *x*)	*x* ( *x*)	*x* ( *x*)	*x* ( *x*)
Missing	*x* ( *x*)	*x* ( *x*)	*x* ( *x*)	*x* ( *x*)	*x* ( *x*)	*x* ( *x*)	*x* ( *x*)	*x* ( *x*)
**Reasons that led mother not to breastfeed baby or not to breastfeed sooner after birth**								
I had a C-section	*x* ( *x*)	*x* ( *x*)	*x* ( *x*)	*x* ( *x*)	*x* ( *x*)	*x* ( *x*)	*x* ( *x*)	*x* ( *x*)
Baby was taken away; baby not well	*x* ( *x*)	*x* ( *x*)	*x* ( *x*)	*x* ( *x*)	*x* ( *x*)	*x* ( *x*)	*x* ( *x*)	*x* ( *x*)
Mother not feeling well	*x* ( *x*)	*x* ( *x*)	*x* ( *x*)	*x* ( *x*)	*x* ( *x*)	*x* ( *x*)	*x* ( *x*)	*x* ( *x*)
Mother did not think the first milk was good	*x* ( *x*)	*x* ( *x*)	*x* ( *x*)	*x* ( *x*)	*x* ( *x*)	*x* ( *x*)	*x* ( *x*)	*x* ( *x*)
Mother said she was not getting milk	*x* ( *x*)	*x* ( *x*)	*x* ( *x*)	*x* ( *x*)	*x* ( *x*)	*x* ( *x*)	*x* ( *x*)	*x* ( *x*)
Cord was not cut, so milk would not come	*x* ( *x*)	*x* ( *x*)	*x* ( *x*)	*x* ( *x*)	*x* ( *x*)	*x* ( *x*)	*x* ( *x*)	*x* ( *x*)
Baby was sleeping	*x* ( *x*)	*x* ( *x*)	*x* ( *x*)	*x* ( *x*)	*x* ( *x*)	*x* ( *x*)	*x* ( *x*)	*x* ( *x*)
Other	*x* ( *x*)	*x* ( *x*)	*x* ( *x*)	*x* ( *x*)	*x* ( *x*)	*x* ( *x*)	*x* ( *x*)	*x* ( *x*)
Do not know	*x* ( *x*)	*x* ( *x*)	*x* ( *x*)	*x* ( *x*)	*x* ( *x*)	*x* ( *x*)	*x* ( *x*)	*x* ( *x*)
**Newborn care during baby’s first month**								
**Did a nurse, doctor, the ASHA or any health worker check on your newborn’s health during the following period of time?**								
**First 3 days**								
Yes	*x* ( *x*)	*x* ( *x*)	*x* ( *x*)	*x* ( *x*)	*x* ( *x*)	*x* ( *x*)	*x* ( *x*)	*x* ( *x*)
No	*x* ( *x*)	*x* ( *x*)	*x* ( *x*)	*x* ( *x*)	*x* ( *x*)	*x* ( *x*)	*x* ( *x*)	*x* ( *x*)
Do not know	*x* ( *x*)	*x* ( *x*)	*x* ( *x*)	*x* ( *x*)	*x* ( *x*)	*x* ( *x*)	*x* ( *x*)	*x* ( *x*)
Baby not alive by then	*x* ( *x*)	*x* ( *x*)	*x* ( *x*)	*x* ( *x*)	*x* ( *x*)	*x* ( *x*)	*x* ( *x*)	*x* ( *x*)
**4th day to 2 weeks**								
Yes	*x* ( *x*)	*x* ( *x*)	*x* ( *x*)	*x* ( *x*)	*x* ( *x*)	*x* ( *x*)	*x* ( *x*)	*x* ( *x*)
No	*x* ( *x*)	*x* ( *x*)	*x* ( *x*)	*x* ( *x*)	*x* ( *x*)	*x* ( *x*)	*x* ( *x*)	*x* ( *x*)
Do not know	*x* ( *x*)	*x* ( *x*)	*x* ( *x*)	*x* ( *x*)	*x* ( *x*)	*x* ( *x*)	*x* ( *x*)	*x* ( *x*)
Baby not alive by then	*x* ( *x*)	*x* ( *x*)	*x* ( *x*)	*x* ( *x*)	*x* ( *x*)	*x* ( *x*)	*x* ( *x*)	*x* ( *x*)
**2–4 weeks**								
Yes	*x* ( *x*)	*x* ( *x*)	*x* ( *x*)	*x* ( *x*)	*x* ( *x*)	*x* ( *x*)	*x* ( *x*)	*x* ( *x*)
No	*x* ( *x*)	*x* ( *x*)	*x* ( *x*)	*x* ( *x*)	*x* ( *x*)	*x* ( *x*)	*x* ( *x*)	*x* ( *x*)
Do not know	*x* ( *x*)	*x* ( *x*)	*x* ( *x*)	*x* ( *x*)	*x* ( *x*)	*x* ( *x*)	*x* ( *x*)	*x* ( *x*)
Baby not alive by then	*x* ( *x*)	*x* ( *x*)	*x* ( *x*)	*x* ( *x*)	*x* ( *x*)	*x* ( *x*)	*x* ( *x*)	*x* ( *x*)
**Checks in all three periods**								
Yes	*x* ( *x*)	*x* ( *x*)	*x* ( *x*)	*x* ( *x*)	*x* ( *x*)	*x* ( *x*)	*x* ( *x*)	*x* ( *x*)
No	*x* ( *x*)	*x* ( *x*)	*x* ( *x*)	*x* ( *x*)	*x* ( *x*)	*x* ( *x*)	*x* ( *x*)	*x* ( *x*)
Do not know	*x* ( *x*)	*x* ( *x*)	*x* ( *x*)	*x* ( *x*)	*x* ( *x*)	*x* ( *x*)	*x* ( *x*)	*x* ( *x*)
Baby not alive by then	*x* ( *x*)	*x* ( *x*)	*x* ( *x*)	*x* ( *x*)	*x* ( *x*)	*x* ( *x*)	*x* ( *x*)	*x* ( *x*)
**Who checked on your newborn’s health during this time?**								
Doctor	*x* ( *x*)	*x* ( *x*)	*x* ( *x*)	*x* ( *x*)	*x* ( *x*)	*x* ( *x*)	*x* ( *x*)	*x* ( *x*)
Auxiliary nurse midwife	*x* ( *x*)	*x* ( *x*)	*x* ( *x*)	*x* ( *x*)	*x* ( *x*)	*x* ( *x*)	*x* ( *x*)	*x* ( *x*)
ASHA	*x* ( *x*)	*x* ( *x*)	*x* ( *x*)	*x* ( *x*)	*x* ( *x*)	*x* ( *x*)	*x* ( *x*)	*x* ( *x*)
Traditional birth attendant	*x* ( *x*)	*x* ( *x*)	*x* ( *x*)	*x* ( *x*)	*x* ( *x*)	*x* ( *x*)	*x* ( *x*)	*x* ( *x*)
Anganwadi worker	*x* ( *x*)	*x* ( *x*)	*x* ( *x*)	*x* ( *x*)	*x* ( *x*)	*x* ( *x*)	*x* ( *x*)	*x* ( *x*)
Registered medical practitioner (RMP)	*x* ( *x*)	*x* ( *x*)	*x* ( *x*)	*x* ( *x*)	*x* ( *x*)	*x* ( *x*)	*x* ( *x*)	*x* ( *x*)
Bengali doctor	*x* ( *x*)	*x* ( *x*)	*x* ( *x*)	*x* ( *x*)	*x* ( *x*)	*x* ( *x*)	*x* ( *x*)	*x* ( *x*)
Other	*x* ( *x*)	*x* ( *x*)	*x* ( *x*)	*x* ( *x*)	*x* ( *x*)	*x* ( *x*)	*x* ( *x*)	*x* ( *x*)
Do not know	*x* ( *x*)	*x* ( *x*)	*x* ( *x*)	*x* ( *x*)	*x* ( *x*)	*x* ( *x*)	*x* ( *x*)	*x* ( *x*)
Missing	*x* ( *x*)	*x* ( *x*)	*x* ( *x*)	*x* ( *x*)	*x* ( *x*)	*x* ( *x*)	*x* ( *x*)	*x* ( *x*)
**During these visits, given counselling on breast feeding?**								
Yes	*x* ( *x*)	*x* ( *x*)	*x* ( *x*)	*x* ( *x*)	*x* ( *x*)	*x* ( *x*)	*x* ( *x*)	*x* ( *x*)
No	*x* ( *x*)	*x* ( *x*)	*x* ( *x*)	*x* ( *x*)	*x* ( *x*)	*x* ( *x*)	*x* ( *x*)	*x* ( *x*)
Do not know	*x* ( *x*)	*x* ( *x*)	*x* ( *x*)	*x* ( *x*)	*x* ( *x*)	*x* ( *x*)	*x* ( *x*)	*x* ( *x*)
Missing	*x* ( *x*)	*x* ( *x*)	*x* ( *x*)	*x* ( *x*)	*x* ( *x*)	*x* ( *x*)	*x* ( *x*)	*x* ( *x*)
**During these visits, given information on keeping baby warm?**								
Yes	*x* ( *x*)	*x* ( *x*)	*x* ( *x*)	*x* ( *x*)	*x* ( *x*)	*x* ( *x*)	*x* ( *x*)	*x* ( *x*)
No	*x* ( *x*)	*x* ( *x*)	*x* ( *x*)	*x* ( *x*)	*x* ( *x*)	*x* ( *x*)	*x* ( *x*)	*x* ( *x*)
Do not know	*x* ( *x*)	*x* ( *x*)	*x* ( *x*)	*x* ( *x*)	*x* ( *x*)	*x* ( *x*)	*x* ( *x*)	*x* ( *x*)
Missing	*x* ( *x*)	*x* ( *x*)	*x* ( *x*)	*x* ( *x*)	*x* ( *x*)	*x* ( *x*)	*x* ( *x*)	*x* ( *x*)
**During these visits, was there an examination of the baby?**								
Yes	*x* ( *x*)	*x* ( *x*)	*x* ( *x*)	*x* ( *x*)	*x* ( *x*)	*x* ( *x*)	*x* ( *x*)	*x* ( *x*)
No	*x* ( *x*)	*x* ( *x*)	*x* ( *x*)	*x* ( *x*)	*x* ( *x*)	*x* ( *x*)	*x* ( *x*)	*x* ( *x*)
Do not know	*x* ( *x*)	*x* ( *x*)	*x* ( *x*)	*x* ( *x*)	*x* ( *x*)	*x* ( *x*)	*x* ( *x*)	*x* ( *x*)
Missing	*x* ( *x*)	*x* ( *x*)	*x* ( *x*)	*x* ( *x*)	*x* ( *x*)	*x* ( *x*)	*x* ( *x*)	*x* ( *x*)
**During these visits, given counselling on child’s immunisation?**								
Yes	*x* ( *x*)	*x* ( *x*)	*x* ( *x*)	*x* ( *x*)	*x* ( *x*)	*x* ( *x*)	*x* ( *x*)	*x* ( *x*)
No	*x* ( *x*)	*x* ( *x*)	*x* ( *x*)	*x* ( *x*)	*x* ( *x*)	*x* ( *x*)	*x* ( *x*)	*x* ( *x*)
Do not know	*x* ( *x*)	*x* ( *x*)	*x* ( *x*)	*x* ( *x*)	*x* ( *x*)	*x* ( *x*)	*x* ( *x*)	*x* ( *x*)
Missing	*x* ( *x*)	*x* ( *x*)	*x* ( *x*)	*x* ( *x*)	*x* ( *x*)	*x* ( *x*)	*x* ( *x*)	*x* ( *x*)
**During these visits, given information on identifying danger signs in the newborn?**								
Yes	*x* ( *x*)	*x* ( *x*)	*x* ( *x*)	*x* ( *x*)	*x* ( *x*)	*x* ( *x*)	*x* ( *x*)	*x* ( *x*)
No	*x* ( *x*)	*x* ( *x*)	*x* ( *x*)	*x* ( *x*)	*x* ( *x*)	*x* ( *x*)	*x* ( *x*)	*x* ( *x*)
Do not know	*x* ( *x*)	*x* ( *x*)	*x* ( *x*)	*x* ( *x*)	*x* ( *x*)	*x* ( *x*)	*x* ( *x*)	*x* ( *x*)
Missing	*x* ( *x*)	*x* ( *x*)	*x* ( *x*)	*x* ( *x*)	*x* ( *x*)	*x* ( *x*)	*x* ( *x*)	*x* ( *x*)
**During these visits, given advice on cord stump care?**								
Yes	*x* ( *x*)	*x* ( *x*)	*x* ( *x*)	*x* ( *x*)	*x* ( *x*)	*x* ( *x*)	*x* ( *x*)	*x* ( *x*)
No	*x* ( *x*)	*x* ( *x*)	*x* ( *x*)	*x* ( *x*)	*x* ( *x*)	*x* ( *x*)	*x* ( *x*)	*x* ( *x*)
Do not know	*x* ( *x*)	*x* ( *x*)	*x* ( *x*)	*x* ( *x*)	*x* ( *x*)	*x* ( *x*)	*x* ( *x*)	*x* ( *x*)
Missing	*x* ( *x*)	*x* ( *x*)	*x* ( *x*)	*x* ( *x*)	*x* ( *x*)	*x* ( *x*)	*x* ( *x*)	*x* ( *x*)

^a^Information for babies was combined using a weighted average (with information from singleton babies who survived the neonatal period given ten times the weight of others)

**Table 12:** Mothers’ knowledge and attitudes (P1) (number (%) unless otherwise stated)

	Mothers of singleton babies who were alive at 28 days		Mothers of multiple birth babies who were all alive at 28 days		Mothers of a baby who was dead at 28 days		Weighted average of all babies^a^	
	CHAMPION2 intervention arm	STRIPES2 intervention arm	CHAMPION2 intervention arm	STRIPES2 intervention arm	CHAMPION2 intervention arm	STRIPES2 intervention arm	CHAMPION2 intervention arm	STRIPES2 intervention arm
**Knowledge of pregnancy danger signs**								
Vaginal bleeding	*x* ( *x*)	*x* ( *x*)	*x* ( *x*)	*x* ( *x*)	*x* ( *x*)	*x* ( *x*)	*x* ( *x*)	*x* ( *x*)
Abdominal pain	*x* ( *x*)	*x* ( *x*)	*x* ( *x*)	*x* ( *x*)	*x* ( *x*)	*x* ( *x*)	*x* ( *x*)	*x* ( *x*)
Severe headache	*x* ( *x*)	*x* ( *x*)	*x* ( *x*)	*x* ( *x*)	*x* ( *x*)	*x* ( *x*)	*x* ( *x*)	*x* ( *x*)
Convulsion	*x* ( *x*)	*x* ( *x*)	*x* ( *x*)	*x* ( *x*)	*x* ( *x*)	*x* ( *x*)	*x* ( *x*)	*x* ( *x*)
Blurred vision	*x* ( *x*)	*x* ( *x*)	*x* ( *x*)	*x* ( *x*)	*x* ( *x*)	*x* ( *x*)	*x* ( *x*)	*x* ( *x*)
Swelling of feet/face/hands	*x* ( *x*)	*x* ( *x*)	*x* ( *x*)	*x* ( *x*)	*x* ( *x*)	*x* ( *x*)	*x* ( *x*)	*x* ( *x*)
Fever	*x* ( *x*)	*x* ( *x*)	*x* ( *x*)	*x* ( *x*)	*x* ( *x*)	*x* ( *x*)	*x* ( *x*)	*x* ( *x*)
Decreased/no foetal movements	*x* ( *x*)	*x* ( *x*)	*x* ( *x*)	*x* ( *x*)	*x* ( *x*)	*x* ( *x*)	*x* ( *x*)	*x* ( *x*)
Foul smelling vaginal discharge	*x* ( *x*)	*x* ( *x*)	*x* ( *x*)	*x* ( *x*)	*x* ( *x*)	*x* ( *x*)	*x* ( *x*)	*x* ( *x*)
Difficulty seeing at night	*x* ( *x*)	*x* ( *x*)	*x* ( *x*)	*x* ( *x*)	*x* ( *x*)	*x* ( *x*)	*x* ( *x*)	*x* ( *x*)
Difficulty in emptying the bladder	*x* ( *x*)	*x* ( *x*)	*x* ( *x*)	*x* ( *x*)	*x* ( *x*)	*x* ( *x*)	*x* ( *x*)	*x* ( *x*)
Feeling weak/feeling tired/breathlessness	*x* ( *x*)	*x* ( *x*)	*x* ( *x*)	*x* ( *x*)	*x* ( *x*)	*x* ( *x*)	*x* ( *x*)	*x* ( *x*)
Water leak	*x* ( *x*)	*x* ( *x*)	*x* ( *x*)	*x* ( *x*)	*x* ( *x*)	*x* ( *x*)	*x* ( *x*)	*x* ( *x*)
**Knowledge of newborn danger signs**								
Poor sucking or feeding	*x* ( *x*)	*x* ( *x*)	*x* ( *x*)	*x* ( *x*)	*x* ( *x*)	*x* ( *x*)	*x* ( *x*)	*x* ( *x*)
Fast or difficult breathing	*x* ( *x*)	*x* ( *x*)	*x* ( *x*)	*x* ( *x*)	*x* ( *x*)	*x* ( *x*)	*x* ( *x*)	*x* ( *x*)
Feels cold or too hot	*x* ( *x*)	*x* ( *x*)	*x* ( *x*)	*x* ( *x*)	*x* ( *x*)	*x* ( *x*)	*x* ( *x*)	*x* ( *x*)
Difficult to wake/lethargic/unconscious	*x* ( *x*)	*x* ( *x*)	*x* ( *x*)	*x* ( *x*)	*x* ( *x*)	*x* ( *x*)	*x* ( *x*)	*x* ( *x*)
Excessive crying	*x* ( *x*)	*x* ( *x*)	*x* ( *x*)	*x* ( *x*)	*x* ( *x*)	*x* ( *x*)	*x* ( *x*)	*x* ( *x*)
Redness of skin around cord/foul smelling discharge	*x* ( *x*)	*x* ( *x*)	*x* ( *x*)	*x* ( *x*)	*x* ( *x*)	*x* ( *x*)	*x* ( *x*)	*x* ( *x*)
Blue skin colour	*x* ( *x*)	*x* ( *x*)	*x* ( *x*)	*x* ( *x*)	*x* ( *x*)	*x* ( *x*)	*x* ( *x*)	*x* ( *x*)
Jaundice	*x* ( *x*)	*x* ( *x*)	*x* ( *x*)	*x* ( *x*)	*x* ( *x*)	*x* ( *x*)	*x* ( *x*)	*x* ( *x*)
No or delayed cry at birth	*x* ( *x*)	*x* ( *x*)	*x* ( *x*)	*x* ( *x*)	*x* ( *x*)	*x* ( *x*)	*x* ( *x*)	*x* ( *x*)
Cough	*x* ( *x*)	*x* ( *x*)	*x* ( *x*)	*x* ( *x*)	*x* ( *x*)	*x* ( *x*)	*x* ( *x*)	*x* ( *x*)
Diarrhoea	*x* ( *x*)	*x* ( *x*)	*x* ( *x*)	*x* ( *x*)	*x* ( *x*)	*x* ( *x*)	*x* ( *x*)	*x* ( *x*)
Vomiting repeatedly	*x* ( *x*)	*x* ( *x*)	*x* ( *x*)	*x* ( *x*)	*x* ( *x*)	*x* ( *x*)	*x* ( *x*)	*x* ( *x*)
Pustules/boils on skin	*x* ( *x*)	*x* ( *x*)	*x* ( *x*)	*x* ( *x*)	*x* ( *x*)	*x* ( *x*)	*x* ( *x*)	*x* ( *x*)
Fits	*x* ( *x*)	*x* ( *x*)	*x* ( *x*)	*x* ( *x*)	*x* ( *x*)	*x* ( *x*)	*x* ( *x*)	*x* ( *x*)
Redness in the eye/infection	*x* ( *x*)	*x* ( *x*)	*x* ( *x*)	*x* ( *x*)	*x* ( *x*)	*x* ( *x*)	*x* ( *x*)	*x* ( *x*)
Congenital anomaly	*x* ( *x*)	*x* ( *x*)	*x* ( *x*)	*x* ( *x*)	*x* ( *x*)	*x* ( *x*)	*x* ( *x*)	*x* ( *x*)
Does not pass stool/urine	*x* ( *x*)	*x* ( *x*)	*x* ( *x*)	*x* ( *x*)	*x* ( *x*)	*x* ( *x*)	*x* ( *x*)	*x* ( *x*)

^a^Information was combined using a weighted average (with information from mothers of singleton babies who survived the neonatal period given ten times the weight of others)

### Analysis methods

Neonatal mortality in this trial has a complex four-level hierarchical structure, with multiple women per cluster, potentially multiple pregnancies per woman, and potentially multiple births per pregnancy. The effect of the active intervention on neonatal mortality (compared to usual care) will be estimated using a generalised estimating equations (GEE) analysis approach. This allows for non-independence of outcomes from the same cluster and for non-independence of multiple outcomes from the same woman. Mixed models (with cluster as a random effect, which are also termed hierarchical or multilevel models) are perhaps more commonly used than GEEs for the analysis of cluster randomised trials. The advantage of the GEE/robust standard error approach here is that the four-level hierarchical structure does not have to be explicitly modelled, so avoiding potential convergence problems.

In detail, the relative risk with a 95% confidence interval will be obtained from a GEE model with a binary outcome, a log link, a “working” assumption of independence with robust standard errors to take account of clustering at village level. The model will include the stratifying variables, which were village size and distance to the nearest community health centre or civil hospital [13, 14].

Secondary analyses will extend the GEE model for the primary outcome described above to (separately) investigate interactions by the randomisation stratifiers, whether women were enrolled pre- or post-randomisation, gender, caste, wealth, and male and female primary caregiver literacy (see below).

The risk difference with a 95% confidence interval will be obtained from a GEE model with a binary outcome, an identity link, a “working” assumption of independence with robust standard errors to take account of clustering. This model will also include the stratifying variables.

Secondary outcomes that are binary will be analysed using the same approach as for the primary outcome.

Maternal mortality in each arm will be expressed as the number of maternal deaths per 100,000 live births, with the ratio of these computed. A nonparametric bootstrap confidence interval (bias corrected and accelerated, 2000 replications at cluster level, stratified by randomisation arm) will be constructed for the ratio (on a log-transformed scale) of these arm-specific mortality rates.

### Adjustment for covariates

All comparisons between trial arms will adjust for the stratification factors (village size and distance to the nearest community health centre or civil hospital (both binary)) and no others.

### Methods used for assumptions to be checked for statistical methods

The models used for the continuous outcomes assume that residuals are normally distributed. Robust standard errors allow for potential heteroscedasticity according to levels of predictor variables but do make an assumption of normality conditional on levels of predictor variables. This assumption will be checked by examination of appropriate quantile-quantile plots of standardised residuals. The central limit theorem ensures that results are robust provided that violations of the normality assumptions are not substantial. Minor violations, even if statistically significant, are of little practical consequence. For this reason, formal hypothesis tests of normality assumptions will not be carried out.

### Alternative methods to be used if distributional assumptions do not hold

Nonparametric bootstrap confidence intervals (bias corrected and accelerated, 2000 replications at cluster level, stratified by randomisation arm) will be reported if the normality assumptions are seriously violated.

### Sensitivity analyses for each outcome where applicable

In the primary analysis, missing data will not be imputed. In secondary analyses of the primary outcome and key secondary outcomes, multiple imputation by chained equations (MICE) will be used if missingness is greater than 5%, as has been recommended [15]. For analysis of clustered data, it is important that the model for imputation includes cluster-specific random effects [16]. Such analyses will be carried out using the Jomo package within the statistical software environment R [17]. Imputation will be carried out separately in each trial arm. Auxiliary variables to potentially be used will include the randomisation stratification factors, caste, gender, male and female primary caregiver literacy and education, the wealth indices, the adherence to intervention variables defined above, being a twin, and previous miscarriage/termination/stillbirth/neonatal death.

If the effect of the intervention is statistically significant, and remains so in the MICE analysis detailed above, then the multiple imputation analysis will also be extended to determine the amount of bias over and above that allowed for by the multiple imputation model that would render the primary analysis non- statistically significant.

### Subgroup analyses

We will conduct exploratory subgroup analyses of the primary outcome by:

- Village population (binary, as used in the stratified randomisation),
- Distance to nearest community health centre/civil hospital (binary, as used in the stratified randomisation),
- Whether women were enrolled pre- or post-randomisation,
- Gender,
- Caste,
- Wealth index 1 (in three categories determined by the material the house is made of),
- Wealth index 2 (in five categories determined by the number of relevant items owned by the household, with the interaction tested using a trend test).
- Primary female caregiver literacy in 3 groups. This to be replaced by female education if more than 10% of the participants have a missing value for literacy and education status is not missing,
- Primary male caregiver literacy in 3 groups. This to be replaced by male education if more than 10% of the participants have a missing value for literacy and education status is not missing.

For each of the above factors, statistical tests for interaction will be carried out, with claims of different effects in subgroups only made if there is strong evidence (*p* < 0.01) of an interaction. See Table 6.

### Additional analyses

The risk difference (and its 95% confidence interval) will be multiplied by the number of live births in the intervention arm to give an estimate (and 95% confidence interval) for the number of lives saved.

Additional analyses will include an economic evaluation. A cost-effectiveness calculation in terms of cost per neonatal death averted and cost per life year saved will be conducted (cost per disability-adjusted life year saved will not be considered as no measure of future disability is available). The sensitivity of these outcomes to the most important inputs—labour costs and exchange rate movements—will be examined.

The direct additional provider costs of the CHAMPION intervention activities compared to existing standard of care in the control arm will be calculated. Total spending will be cross-checked with funding sources for accuracy. Equipment and other resources provided to clinics which benefitted both control and intervention villages will be noted separately. Spending will be divided into running costs and capital costs. Start-up costs are limited and are assumed to be fully depreciated during the trial because the NICE Foundation had previously implemented a similar version of the programme elsewhere in India. Straight line depreciation of capital equipment (computers, ambulances, and medical equipment will be based on 3-, 4-, and 8-year lifespans, respectively) will be allowed for, consistent with usual account practices. Capital spending outside these items is assumed to be fully depreciated immediately. There are no contributions in kind.

Annual cost figures will be adjusted by India’s GDP deflator in order to convert values to July 2023 rupees. Average exchange rates from July 2023 will be used to convert rupee figures to US dollars.

### Statistical software

Stata version 18 (StataCorp. 2023. Stata Statistical Software: Release 18. College Station, TX: StataCorp LLC) and/or R (R Core Team 2022. R: A language and environment statistical computing. R Foundation for Statistical Computing, Vienna, Austria. URL https://www.R-project.org/.)

## Trial status and declarations

### Trial status

The statistical analysis plan is based on the published protocol [8]. Its content and structure follow a recommended checklist (see supplementary material). 

This is a cluster randomised trial, with all villages (clusters) randomised in 2019. Eligible women for the CHAMPION2 trial were enrolled at enumeration (pre-randomisation) and after this (post randomisation) if marrying a man who was enumerated and unmarried at the time of enumeration or who was enumerated and had subsequently become widowed. Data collection finished soon after the final births, which were on 30 June 2023. Data cleaning for CHAMPION2 is ongoing prior to anticipated data-lock in April 2024.

### Data management plan

The database has been developed by Sealed Envelope (https://www.sealedenvelope.com), an independent company contracted to construct and maintain a bespoke database for the trial who will also keep a periodical backup of the data.

### Trial master file, statistical master file, and standard operating procedures

The trial master file is part of the standard operating procedures manual. The standard operating procedures manual is available upon request. The statistical master file is held securely and may be available upon request after final analyses.

### Supplementary Information

**Supplementary Material 1.**

## Data Availability

Data sharing is not applicable to this article (a statistical analysis plan) as no datasets will be generated or analysed during this stage of the study. After publication of the initial results, the anonymised datasets used and/or analysed during the trial with relevant statistical code will be available from the corresponding author on reasonable request.
