# Supplementary material for: Statistical analysis plan for a cluster randomised trial in Madhya Pradesh, India: community health promotion and medical provision and impact on neonates (CHAMPION2)
Source: Trials. 2024 Apr 25;25:280. doi: 10.1186/s13063-024-08056-2 (PMC11045454; doi:10.1186/s13063-024-08056-2)
Supplement: Supplementary file 1 — Supplementary Material 1. [file 13063_2024_8056_MOESM1_ESM.docx]

**Statistical Analysis Plan (SAP) Checklist v 1.0 2019**

| Section/Item | Index | Description | Reported on page # |
| --- | --- | --- | --- |
| **Section 1: Administrative information** | | | |
| Trial and Trial registration | 1a | Descriptive title that matches the protocol, with SAP either as a forerunner or subtitle,  and trial acronym (if applicable) | 1 |
|  | 1b | Trial registration number | 1 |
| SAP Version | 2 | SAP version number with dates | 1 |
| Protocol Version | 3 | Reference to version of protocol being used | 1 |
| SAP revisions | 4a | SAP revision history | N/A |
|  | 4b | Justification for each SAP revision | N/A |
|  | 4c | Timing of SAP revisions in relation to interim analyses, etc. | N/A |
| Roles and responsibility | 5 | Names, affiliations, and roles of SAP contributors | 1 and 2 |
| Signatures of: | 6a | Person writing the SAP | 1 |
|  | 6b | Senior statistician responsible | 1 |
|  | 6c | Chief investigator/clinical lead | 1 |
| **Section 2: Introduction** | | | |
| Background and rationale | 7 | Synopsis of trial background and rationale including a brief description of research question  and brief justification for undertaking the trial | 4 and 5 |
| Objectives | 8 | Description of specific objectives or hypotheses | 5 |
| **Section 3: Study Methods** | | | |
| Trial design | 9 | Brief description of trial design including type of trial (e.g., parallel group, multi-arm, crossover, factorial)  and allocation ratio and may include brief description of interventions | 5-7 |
| Randomization | 10 | Randomization details, e.g., whether any minimization or stratification occurred (including stratifying factors used or the location of that information if it is not held within the SAP) | 7 |
| Sample size | 11 | Full sample size calculation or reference to sample size calculation in protocol  (instead of replication in SAP) | 7 and 8 |
| Framework | 12 | Superiority, equivalence, or noninferiority hypothesis testing framework, including which comparisons will be presented on this basis | 9 |
| Statistical interim analysis and stopping guidance | 13a | Information on interim analyses specifying what interim analyses will be carried out  and listing of time points | 9 |
|  | 13b | Any planned adjustment of the significance level due to interim analysis | 9 |
|  | 13c | Details of guidelines for stopping the trial early | 9 |
| Timing of final analysis | 14 | Timing of final analysis, e.g., all outcomes analysed collectively or timing stratified  by planned length of follow-up | 9 |
| Timing of outcome assessments | 15 | Time points at which the outcomes are measured including visit “windows” | 9 and 10 |
| **Section 4: Statistical Principals** | | | |
| Confidence intervals and *P* values | 16 | Level of statistical significance | 10 |
|  | 17 | Description and rationale for any adjustment for multiplicity and, if so, detailing how the type 1 error is to be controlled | 10 |
|  | 18 | Confidence intervals to be reported | 10 |
| Adherence and Protocol deviations | 19a | Definition of adherence to the intervention and how this is assessed including extent  of exposure | 10 and 11 |
|  | 19b | Description of how adherence to the intervention will be presented | 11 and 12 |
|  | 19c | Definition of protocol deviations for the trial | 13 |
|  | 19d | Description of which protocol deviations will be summarized | 13 |
| Analysis populations | 20 | Definition of analysis populations, e.g., intention to treat, per protocol,  complete case, safety | 13 and 14 |
| **Section 5: Trial Population** | | | |
| Screening data | 21 | Reporting of screening data (if collected) to describe representativeness  of trial sample | N/A |
| Eligibility | 22 | Summary of eligibility criteria | 15 |
| Recruitment | 23 | Information to be included in the CONSORT flow diagram | 16 |
| Withdrawal/ Follow-up | 24a | Level of withdrawal, e.g., from intervention and/or from follow-up | 16 and 17 |
|  | 24b | Timing of withdrawal/lost to follow-up data | 16 and 17 |
|  | 24c | Reasons and details of how withdrawal/lost to follow-up data will be presented | 16 and 17 |
| Baseline patient characteristics | 25a | List of baseline characteristics to be summarized | 17 and 18 |
|  | 25b | Details of how baseline characteristics will be descriptively summarized | 18 to 20 |
| **Section 6: Analysis** | | | |
| Outcome definitions |  | List and describe each primary and secondary outcome including details of: |  |
|  | 26a | Specification of outcomes and timings. If applicable include the order of importance of primary  or key secondary end points (e.g., order in which they will be tested) | 20 to 47 |
|  | 26b | Specific measurement and units (e.g., glucose control, hbA1c [mmol/mol or %]) | 20 to 47 |
|  | 26c | Any calculation or transformation used to derive the outcome (e.g., change from baseline, QoL score, time to event, logarithm, etc.) | 20 to 47 |
| Analysis methods | 27a | What analysis method will be used and how the treatment effects will be presented | 48 to 52 |
|  | 27b | Any adjustment for covariates | 49 |
|  | 27c | Methods used for assumptions to be checked for statistical methods | 49 |
|  | 27d | Details of alternative methods to be used if distributional assumptions do not hold, e.g., normality, proportional hazards, etc. | 49 |
|  | 27e | Any planned sensitivity analyses for each outcome where applicable | 50 |
|  | 27f | Any planned subgroup analyses for each outcome including how subgroups are defined | 50 and 51 |
| Missing data | 28 | Reporting and assumptions/statistical methods to handle missing data (e.g., multiple imputation) | 50 |
| Additional analyses | 29 | Details of any additional statistical analyses required, e.g., complier-average causal effect analysis | 51 and 52 |
| Harms | 30 | Sufficient detail on summarizing safety data, e.g., information on severity, expectedness, and causality; details of how adverse events are coded or categorized; how adverse event data will be analysed, i.e., grade 3/4 only, incidence case analysis, intervention emergent analysis | 28 and 29 |
| Statistical software | 31 | Details of statistical packages to be used to carry out analyses | 52 |
| References | 32a | References to be provided for nonstandard statistical methods | N/A |
|  | 32b | Reference to Data Management Plan | 53 |
|  | 32c | Reference to the Trial Master File and Statistical Master File | 53 |
|  | 32d | Reference to other standard operating procedures or documents to be adhered to | 53 |

**Taken from the paper:** Gamble C, Krishan A, Stocken D, Lewis S, Juszczak E, Doré C, et al. Guidelines for the Content of Statistical Analysis Plans in Clinical Trials. JAMA. 2017;318(23):2337-43.

**Abbreviations:** CONSORT, Consolidated Standards of Reporting Trials; hbA1c, haemoglobin A1c; QoL, quality of life; SAP, statistical analysis plan.

For more information visit:

*The development of this checklist was funded by the* [*MRC Hubs for Trials Methodology Research*](https://www.methodologyhubs.mrc.ac.uk/)
